# On optimal and near-optimal shapes of external shading of windows in apartment buildings

**DOI:** 10.1371/journal.pone.0212710

**Published:** 2019-02-28

**Authors:** Sanja Stevanović, Dragan Stevanović, Matthias Dehmer

**Affiliations:** 1 Mathematical Institute, Serbian Academy of Sciences and Arts, Belgrade, Serbia; 2 Institute for Intelligent Production, Faculty for Management, University of Applied Sciences Upper Austria, Steyr Campus, Steyr, Austria; 3 College of Computer and Control Engineering, Nankai University, Tianjin, PR China; University of Science and Technology Beijing, CHINA

## Abstract

We studied previously optimal shape of external shading of windows in a cellular office with an outer edge modeled by a non-uniform rational basis spline (NURBS) curve whose control points were placed uniformly around western fin, overhang and eastern fin of the window, and whose depths were allowed to vary independently. We observed there that for each climate considered in the study there exists a shading shape close to the optimal one, but with a substantially simpler structure of control points for the NURBS curve. This simpler structure was reflected in partitioning control points into six groups such that all control points in the same group have equal depths, with groups corresponding to lower part of the western fin, upper part of the western fin, joint of the western fin and the overhang, internal part of the overhang, joint of the overhang and the eastern fin and the remaining part of the eastern fin. Here we confirm that shadings with control point structure restricted in such way can perform as well as shadings with unrestricted control points by optimising shape of external shading of windows in an apartment room for both restricted and unrestricted control point structure for the same range of climates, and showing that differences in heating and cooling demands between Pareto optimal shadings in both cases are negligibly small. This grouping of control points thus gives a simple and natural division of shading into a small number of basic constituents that have most impact on its heating and cooling demands. We further consider the convex hull of the Pareto front for shadings with restricted control points, as it contains shadings that minimise equivalent source energy in terms of the ratio of efficiencies and source energy conversion factors for district heating and cooling. We show that, in cases when depths of control point groups in convex hull shadings do not experience sudden changes between their extremal values, these depths can be fitted reasonably well by a sigmoid function that results in functional shadings that satisfactorily approximate heating and cooling demands of shadings in the Pareto front.

## Introduction

### Literature review

Shading of windows is a standard way for passive reduction of cooling loads in buildings, popularised by Le Corbusier in the form of brise-soleils in his Unité d’habitation buildings in the 1950s [1]. Older exterior shading design methods [2, 3] were mainly inspired by Le Corbusier’s goal of fully shading windows at noon in summer, without blocking the sun in winter. These methods assume that overheating in buildings is directly related to incidence of solar radiation on windows. Solar paths are projected either on a horizontal plane [2] or a vertical cylinder [3], and building overheating periods are then plotted on such solar path diagrams. This helps to define a shading mask that avoids direct solar radiation during these periods, which, when projected back to a building, defines an appropriate shading element. This process involves nonlinear projections that cannot be easily performed with manual drawing tools, so that several pieces of software were developed to teach and automatize it (see, e.g., [4, 5]).

Interesting variations on previous theme are presented in [6–8] that propose geometrical methods to determine shading shape by tracing back solar rays at specified cut-off days and cut-off times. A method by Arumí-Noé [6] for a selected winter design day traces back solar rays from window edges and corners to determine funnel surface that ensures that the window is never shaded during the winter design day, and then uses solar rays on a summer design day to clip the funnel surface so that the window is fully shaded during the summer design day. Marsh’s method [7] determines the shading that will completely shade the window during specified summer cut-off dates and times. It starts with a surface that will contain the shading and traces solar rays from the two lower vertices of the window sill back to the surface. The resulting shading is formed by projections of a morning analemma on the east and an afternoon analemma on the west at specified cut-off times, and parts of two solar paths at specified cut-off date between the cut-off times. While such form does completely shade the window during specified times, it easily results in excessive area unless the shading surface is placed relatively tightly around all window sides. Somewhat similar is a method by Gupta and Ralegaonkar [8] that determines shading for hot and dry climates by tracing back solar rays for two specified cut-off dates: a winter cut-off date after which shading should allow full entry of solar radiation through the window and a summer cut-off date, and cut-off times, after which solar radiation should be fully blocked. In this method, shading area is restricted by specifying maximum distance of the shading from the building façade and maximum extension beyond each side and above the window.

Confronted with restricted availability of daylight in densely-packed urban environments such as Hong Kong, Cheung and Chung [9] divide the hemispherical sky into 5° × 5° patches, determine from long-term climate data the probable sunlight duration when sun is found in a given patch and plot sunlight duration data on a cylindrical sky map. Curves that represent projections of overhangs and sidefins of various dimension ratios with respect to window width and height are also plotted on the sky map, helping the designer to visualise sunlight duration data and adequately adapt the shading design.

Previous methods deal with the incidence of solar radiation on window surface only, while the amount of direct solar radiation transmitted into the building through fenestration depends on both the cosine of the incidence angle and glazing g-value. To correct this, Dubois [10] improved Mazria’s solar path diagrams to further include information on the intensity of solar radiation and graphical representation of a multiplicative factor that determines the amount of transmitted radiation, which enables the designer to more realistically choose critical cut-off dates and times for shading, thus avoiding oversized shadings. A very interesting related digression is contained in [11] where the authors have used the same information as Dubois in order to quantify shadow patterns of a given horizontal shading device and perform an opposite task: to design the curved window to fit a shading device instead of designing the shading device to fit a window…

A more recent group of shading methods [12–14] proceeds by dividing the support structure of shading into a two-dimensional array of cells and calculating the effect that each individual cell has on heating and cooling demands. These methods produce ordering of surface cells from the most to the least effective ones, which helps the designer to fit the whole shading into a given surface area. For each cell and each hour, Kaftan’s cellular method [12] checks whether direct solar radiation reaching a window is passing through a given cell, calculates the amount of solar radiation during that hour that would enter the room if non-shaded by a cell (or the amount that would be eliminated from the room if shaded by a cell), and compare these results with the results of energy and daylight simulations under the assumption that the window is fully shaded at all times, to determine if shading is beneficial or undesirable during that particular hour. The cellular method had been integrated with thermal simulation engine into Ecotect [13], which enables its easy application in AutoCAD. Shaderade method [14] calculates sensible heating and cooling loads for a space without an external shading system, and for each time segment declares undesirable that quantity of transmitted direct solar radiation that is equal to the difference between cooling and heating loads during that time segment. Then for each cell, its transmittance is calculated as a value that minimizes undesirable radiation over all time periods when the cell shades a part of the window. This method had been coupled with EnergyPlus and implemented it in Rhinoceros.

Despite the fact that fixed shading devices reduce available daylight in adjacent spaces, previous shading design methods were dealing mainly with heating and cooling loads, probably because the importance of daylighting for a number of human physiological factors started to get understood only less than twenty years ago [15, 16]. An early example of research related to both glare issues of daylighting is provided by Yener [17], who described a procedure to calculate daylighting glare index under the presence of horizontal shading device, and who mostly confirmed that Olgyay’s method does not lead to high values of daylighting glare index. Nabil and Mardaljević [18] argued that widely used daylight factor is not sensitive enough to describe daylighting performance, and suggested the useful daylight illuminance index instead, that gives the percentage of time periods during which illuminance provided by daylight lies in a given range, so that it is not too dim for work and not too high to produce glare. Based on a review of several published studies, Nabil and Mardaljević suggested the range 100–2000 lux as useful illuminance range, although also other ranges may be found in the literature (for example, 300–8000 lux in [19]).

To accommodate both thermal and daylighting objectives, newer shading studies usually turn to optimization algorithms to produce satisfactory designs through a number of simulations. Manzan and his coauthors [20–22] set a good example in this way by using genetic algorithms to optimize geometrical parameters of a flat pannel shading device positioned parallel to the window and inclined by its horizontal axis. The optimal shading is defined in [20] and [21] as the one that minimizes annual primary energy consumption, which is a weighted sum of heating, cooling and artificial lighting loads. On the other hand, [22] illustrates the use of genetic algorithms in approximating Pareto fronts by considering two objective functions: the annual primary energy consumption and the number of hours of activation of automated internal shading set up to be fully lowered as soon as direct solar irradiance reaches 50 W/m^2^ on interior sensors (and fully open otherwise). Another example of similar approach was given by Khoroshiltseva et al [23], who used harmony search to approximate Pareto front with respect to three objective functions: length of overheating period, change in energy demands for heating and lighting (case study was located in Madrid, so that cooling was not considered), and area of the shading device.

At the end of this short literature review, let us mention two research reports on overhang shading devices installed in real buildings. Huang et al [24] analyzed fixed overhang shading installed in a university campus in Hong Kong: while it was the least efficient among several alternative shading devices in terms of cooling load reduction, it was at the same time the most reliable in terms of structure, as external shading was expected to withstand extreme weather conditions (typhoons and thunderstorms). Cho et al [25] similarly showed that there are more efficient external shading devices than the overhang, but that for aesthetic reasons and minimum simple payback time, overhang shading devices are most suitable for retrofits in high-rise residential buildings.

### Study goals

We are interested here in the shape of optimal fixed external shading device consisting of western fin, overhang and eastern fin placed tightly around a south-facing window. Curvilinearity of solar paths leads to expectation that optimal shadings may be curvilinear as well, as Kaftan had suggested already in [12]: “Since heat loads and daylight intensities vary according to different sun angles, even a rectangular window will not have an optimal shading form with simple geometry.” To model such curvilinearity here we use NURBS curves to represent the outer edge of shading. In our previous case study [26] we investigated optimal shapes of curvilinear external shading of a window in a cellular office, where depth of each control point was allowed to vary independently. That study, performed for a cellular office placed in the Pacific Northwest National Laboratory (PNNL) office building model for 16 representative USA climates, with the aim of minimizing equivalent source energy needed for heating, cooling and lighting in the cellular office, has reached as one of its conclusions that for each optimal shading found there exists a significantly simpler shading design whose equivalent source energy needs are at most 0.24% higher than the optimal value. These simpler shadings are obtained by partitioning the set of all control points into six groups of consecutive control points such that all control points in the same group have equal depths, with groups corresponding to lower part of the western fin, upper part of the western fin, joint of the western fin and the overhang, internal part of the overhang, joint of the overhang and the eastern fin and the remaining part of the eastern fin. Such grouping of control points provides a simple and natural division of shading into a smaller number of basic constituents, reducing the number of necessary parameters that define shading’s outer edge. Our first goal here is to confirm that shadings with control points restricted in this way can perform as well as shadings with independent control points also in the case of windows in high-rise apartments, which is done by performing two sets of optimisations, for shadings with independent control points and for shadings with grouped control points, for a window of a room in the PNNL high-rise apartment building model for 16 representative USA climates.

Unlike the PNNL office building models, the PNNL apartment building models have no minimal lighting requirements so that window shading influences heating and cooling demands only. Hence, optimal solutions in the case of apartment building model can be explored through their Pareto fronts, and in particular through their convex hulls, which represent shadings with minimal equivalent source energy demand for varying ratios of source energy factors and district heating and cooling efficiencies. Our second goal is to illustrate that depths of control point groups in the convex hull shadings can be fitted by a sigmoid function in terms of this ratio and that, in cases when depths of control point groups in the convex hull shadings do not experience sudden jumps or falls, shadings with fitted depths of control point groups can closely approximate heating and cooling demands of shadings belonging to the Pareto front.

## Methods

The process of optimizing external shading is described in detail in subsequent subsections. To put it succintly, each control point of an outer NURBS curve has a predefined set of positions and the search space consists of NURBS curves determined by all combinations of such control point positions. Genetic algorithm, as implemented in simulation manager jEPlus+EA [27], is instructed to approximate Pareto front of heating and cooling demands of various shading alternatives, which are modeled through auxiliary Python package and simulated by EnergyPlus. For further analysis, convex hull of the approximated Pareto front is found by a local implementation of th Fortune’s variant of the Graham’s scan method [28], while fitting of control point depths in convex hull shadings is done by the Levenberg-Marquardt algorithm [29, 30], as implemented in gnuplot.

To illustrate the optimization method the PNNL high-rise apartment building model [31–33] is chosen here, as it contains realistic settings of building materials and load schedules. In order to simplify modeling, no neighboring buildings or exterior obstacles were considered. Should it be necessary, they can be easily included in the model, as the optimization method is based on simulations with EnergyPlus, which takes exterior obstacles into account in its calculations.

### Apartment room model

The prototype high-rise apartment building models [31–33], developed by PNNL from the Department of Energy (DOE) Commercial Reference Building Models, are used as a starting point in this study. These models represent realistic building characteristics and construction practices, cover more than 80% of the commercial building floor area in the United States for new construction, and conform to the American Society of Heating, Refrigerating and Air-Conditioning Engineers (ASHRAE) 90.1-2013 standard. They are provided for a number of locations that represent main USA climate zones, according to the climate zone classification system of Briggs et al. [34], including Vancouver, Canada which represents the cool, marine climate zone 5C. Their locations, together with a few further selected parameters, are given in Table 1, reproduced from our previous study [26].

**Table 1:** Model locations and selected parameters [26].

Location	Climate zone	Latitude (°N)	Incident solar radiation rate (W/m^2^)	Window U-value (W/m^2^K)	Window SHGC
Miami	1A: very hot, humid	25.82	121.74	0.60	0.25
Houston	2A: hot, humid	30.00	118.41	0.60	0.25
Phoenix	2B: hot, dry	33.45	167.94	0.60	0.25
Memphis	3A: warm, humid	35.07	129.20	0.55	0.25
El Paso	3B: warm, dry	31.77	162.14	0.55	0.25
San Francisco	3C: warm, marine	37.62	141.94	0.55	0.25
Baltimore	4A: mixed, humid	39.17	129.45	0.42	0.40
Albuquerque	4B: mixed, dry	35.04	168.25	0.42	0.40
Salem	4C: mixed, marine	44.90	116.35	0.42	0.40
Chicago	5A: cool, humid	41.98	122.67	0.42	0.40
Boise	5B: cool, dry	43.62	144.32	0.42	0.40
Vancouver	5C: cool, marine	49.18	111.99	0.42	0.40
Burlington	6A: cold, humid	44.47	118.52	0.42	0.40
Helena	6B: cold, dry	46.60	143.85	0.42	0.40
Duluth	7: very cold	46.83	129.84	0.40	0.45
Fairbanks	8: subarctic	64.82	114.49	0.40	0.45

The incident solar radiation rate column gives the average annual solar radiation rate incident to the exterior southern wall surface as returned by EnergyPlus output variable *Surface Outside Face Incident Solar Radiation Rate per Area*.

**Fig 1:**
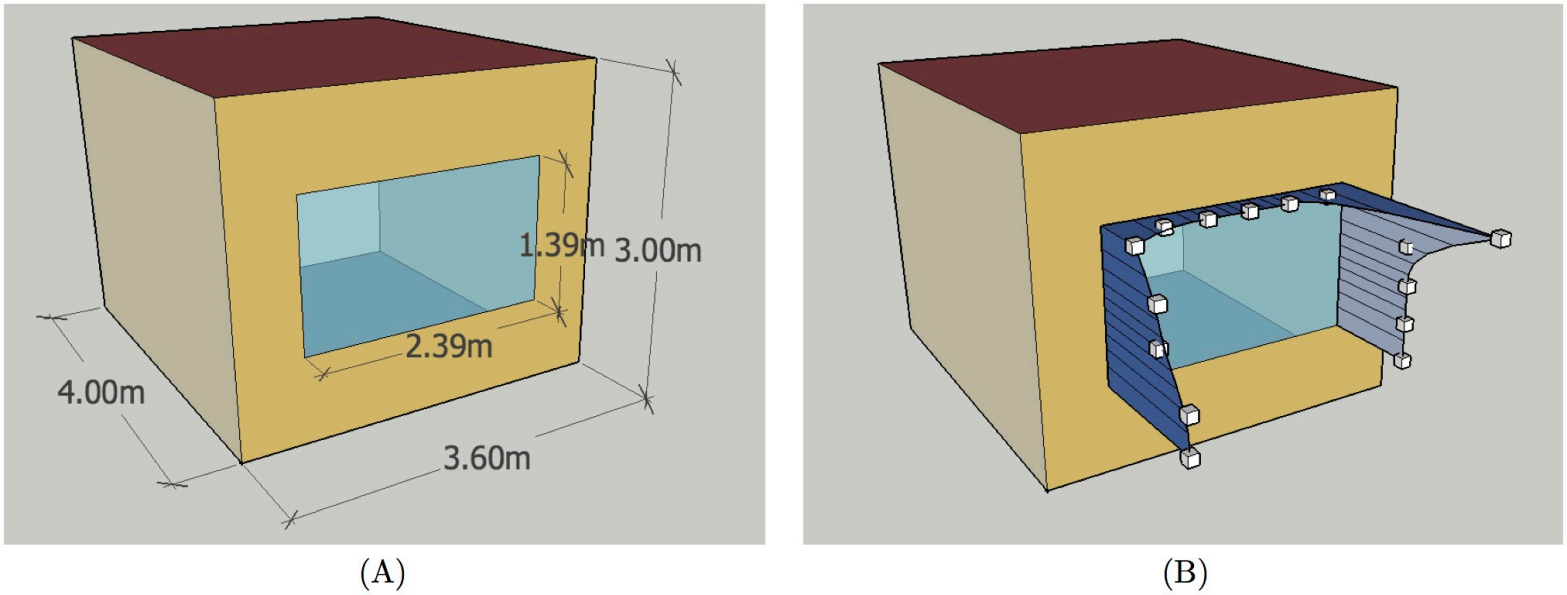
Apartment room model used in simulations: (A) dimensions; (B) small boxes indicate positions of 15 control points of NURBS curves.

Our aim is to study effects of shading a single south-facing window, so that an apartment room has been set up as a single zone, using settings, materials, constructions and schedules of the underlying PNNL models. The room has width 3.6 m, height 3 m and depth 4 m, with the external wall oriented toward south and the other walls assumed to be adiabatic. The window in the room has width 2.39m and height 1.39m, in order to keep the same windows-to-wall ratio as in the PNNL high-rise apartment building model, with the window sill set at 0.8m from the floor. The room model is illustrated in Fig 1A. Window glazing properties depend on climate zone, and their U-values and solar heat gain coefficients are listed in Table 1. Power densities of lighting and electric equipment are, respectively, set to 4.844 W/m^2^ (for both hardwired and plugin lighting) and 6.67 W/m^2^, with actual consumption controlled by separate schedules. Heating and cooling setpoints are, respectively, set to 21.7°C and 24.4°C. Since the simulations are run for a single room instead of the whole building, detailed heating, ventilation and air-conditioning (HVAC) information from the PNNL models had been replaced with *IdealLoadsAirSystem*.

The models were prepared for simulations with EnergyPlus 8.4. To ensure proper calculation of shading effects, the calculation method field of the *ShadowCalculation* object is set to *TimeStepFrequency*, which performs solar path, shadowing and diffuse sky modeling calculations at each 15-minute timestep, while the solar distribution field of the *Building* object is set to *FullInteriorAndExterior*, which computes patterns of shadows cast by the window shading on exterior surfaces and calculates amounts of transmitted beam radiation falling on each internal surface by projecting the sun’s rays through the window. Archive with simulation files for an apartment room model for all locations is available at [35].

### NURBS curves

NURBS is a widely accepted standard in computer-aided design, engineering and manufacturing for describing and generating smooth curves and surfaces [36, 37]. A NURBS curve is defined by a sequence of control points *P*_*i*_, *i* ∈ *I* for some index set *I*, that act as if *P*_*i*_ were connected to the curve by a spring of strength *w*_*i*_. Each point of the NURBS curve *C*(*t*), 0 ≤ *t* ≤ 1, is then a convex combination of control points:

$$\begin{array}{c} C\left(t\right)=\frac{\sum_{i\in I}w_{i}N_{i}\left(t\right)P_{i}}{\sum_{i\in I}w_{i}N_{i}\left(t\right)}, \end{array}$$

where *N*_*i*_(*t*) are suitably calculated basis functions. The basis functions are determined by a degree *d* and a knot vector which partitions the interval [0, 1] into knot spans, in such a way that ∑_*i*∈*I*_ *N*_*i*_(*t*) = 1 holds for each *t* ∈ [0, 1] and that each basis function has *d* + 1 consecutive knot spans on each of which it reduces to a polynomial of degree *d*, while it is equal to zero outside these knot spans. These conditions ensure that each curve point is determined by *d* + 1 closest control points. Details of computation of basis functions may be found in [36, 37].

As in [26], the window shading for the apartment room model consists of three parts: western fin, overhang and eastern fin. They are placed tightly around the window in vertical planes (fins) or in a horizontal plane (overhang), with their outer edges modeled as NURBS curves. As a compromise between the ability to model as many curves as possible and the ability to efficiently run optimisation studies, the number of NURBS control points had to be set to a moderate value. There is a total of 15 control points:

$$\begin{array}{lll} P_{i}=(0.6, & y_{i},\;0.8+0.35i) & \text{for}\;i=0,\ldots ,4,\\ P_{i}=(0.6+0.4(i-4), & y_{i},\;2.2) & \text{for}\;i=4,\ldots ,10,\\ P_{i}=(3.0, & y_{i},\;2.2-0.35(i-10)) & \text{for}\;i=10,\ldots ,14, \end{array}$$

whose coordinates are given with respect to the lower left corner of the outside surface of the exterior wall. The NURBS curve of the western fin is determined by control points *P*_0_, …, *P*_4_, the one of the overhang by control points *P*_4_, …, *P*_10_, and that of the eastern fin by control points *P*_10_, …, *P*_14_. All control points are of unit weight. For each curve the basis functions are of degree three, determined by a clamped uniform knot vector that starts and ends with three empty knot spans, ensuring that the curve starts with its first and ends with its last control point. Due to the common control points *P*_4_ and *P*_10_, the ends of the overhang coincide with the upper ends of fins, giving the shading a continuous look. It should be noted, however, that internal control points of curves do not necessarily belong to them, so that the *y*_*i*_ values do not represent actual shading depths, but only the (negative) distances of control points from the external wall. An example of window shading for the apartment room model, with control points shown as small boxes, is illustrated in Fig 1B.

Additional benefit of using NURBS curves to model outer edge of shading is the possibility to control size of the search space. In the first optimisation study, where the *y*_*i*_ values were set independently of each other for *i* = 0, …, 14, they were prescribed to take values from the set {0, 0.25, 0.5, 0.75, 1, 1.25, 1.5, 1.75, 2}, so that the search space in that case contained 9^15^ ≈ 2.06 ⋅ 10^14^ feasible curves. In the second optimisation study, where the control points were partitioned into six groups, the *y*_*i*_ values were restricted so that

$$\begin{array}{c} y_{0}=y_{1},\;y_{2}=y_{3},\;y_{5}=\cdots =y_{9},\;\text{and}\;y_{11}=\cdots =y_{14}. \end{array}$$

To avoid that this restriction substantially reduces size of the search space, the *y*_*i*_ values were given a higher resolution, so that they could be set between 0m and 2m in 0.01m steps. Consequently, size of the search space in this case was 201^6^ ≈ 6.59 ⋅ 10^13^. To facilitate comparison between the results of these two optimisation studies, in the sequel we will refer to the former as *general* and to the latter as *simplified* optimisation study, where general and simplified describe the two NURBS control point structures used to generate window shadings. The fact that the considered NURBS curves are determined by a small number of parameters makes it possible to search for the optimal shading shape with one of current optimisation methods.

Since EnergyPlus can model building geometry using rectilinear surfaces (with at most four vertices) only and cannot handle NURBS curves directly, we have developed in previous study [26] a Python package epnurbs to overcome this limitation. Its source code is available at [38], while it can be installed with the usual pip install epnurbs. The main method in the package is createnurbsshading that adds an approximation of NURBS lined shading to an arbitrary wall surface in a model: it loads the EnergyPlus model, finds the base surface, divides the domain interval [0, 1] uniformly by points *t*_*i*_ = *i*/*k* for *i* = 0, …, *k* for given *k*, and then calculates NURBS curve points *C*(*t*_*i*_) and their feet of perpendiculars to the base surface. NURBS lined shading is approximated by adding to the model a number of adjacent trapezoidal shadings, where the *i*-th shading, for *i* = 0, …, *k* − 1, has as vertices the curve points *C*(*t*_*i*_), *C*(*t*_*i*+1_) and their projections on the wall surface. Detailed example of the use of createnurbsshading can be found in the archive with simulation files [35].

### Simulation and optimisation management

EnergyPlus simulations for locations mentioned in Table 1 were managed with jEPlus [39–41]. jEPlus enables one to perform parametric EnergyPlus simulations by describing a search space with sets of alternative values for specified simulation parameters and running simulations either for the whole search space or its representative sample. jEPlus also provides the ability to call a Python program to preprocess simulation files, which in this case creates a list of alternative positions of control points, selects the appropriate positions based on current parameter values and calls createnurbsshading from epnurbs to add to the apartment room model the western fin approximated with 10 trapezoids, the overhang approximated with 15 trapezoids and the eastern fin approximated with 10 trapezoids, after which the model is simulated with EnergyPlus 8.4.

Due to prohibitively large search spaces in this study, jEPlus cannot be used directly to search for optimal shading shapes, but has to be coupled with an optimisation method instead. Such coupling had become mainstream in the study of energy and buildings after Caldas and Norford [42] used it prominently to facilitate performance-based façade design, and reviews of current literature on building design optimisation can be found in, e.g., [43–45]. Probably led by Caldas and Norford’s early example, most building design optimisation studies still rely on the use of genetic algorithms. While other optimisation methods had been used in the literature, one could still say that building design optimisation community lacks behind other engineering communities in accepting newer optimisation methods, such as, e.g., Jaya [46–49] or Harmony search [23]. One of the apparent reasons for this is that genetic algorithms tend to work well for building design optimisation problems, while another reason may be that there exists user-friendly free software such as jEPlus+EA [27], coupling EnergyPlus with a well known elitist genetic algorithm variant NSGA-II [50]. jEPlus+EA was used in this study as well, with heating and cooling demands set as objective functions to be optimised.

In the general optimisation study, where a NURBS lined shading has independently set control points, *N*_*b*,1_ = 15 ⋅ 4 = 60 bits are needed to describe its parameter values (4 bits to denote each possible depth from the set {0, 0.25, …, 2}). Taking into account Gutowski’s recommendations obtained from his linear algebraic treatment of genetic algorithms [51], population size was set to *N*_1_ = 16, slightly larger than the recommended minimal size $\left\lceil \sqrt{2N_{b,1}}\right\rceil$, while crossover rate was set at 0.8 and mutation rate at $p_{mut,1}=3.3\cdot 10^{-3}\approx 1-0.82^{1/N_{b,1}}$. Such population is expected to become mature after $\frac{1}{2p_{mut,1}}\approx 150$ generations [51], and we stopped genetic optimisation after it reached at least 400 generations.

In the simplified optimisation study, where a NURBS lined shading has control points divided in groups with equal depths, *N*_*b*,2_ = 6 ⋅ 8 = 48 bits are needed to describe its parameter values (8 bits to denote each depth, which is an integer between 0 and 201). Population size was set to *N*_2_ = 16 as in the first case. Mutation rate was set at $p_{mut,2}=4.1\cdot 10^{-3}\approx 1-0.82^{1/N_{b,2}}$, while crossover rate was set at 0.8. In this case the population would become mature after $\frac{1}{2p_{mut,2}}\approx 120$ generations, and genetic optimisation was similarly stopped after it went through at least 400 generations.

### Pareto front and its convex hull

For each shading variant *S*(*y*_0_, …, *y*_14_) considered by the NSGA-II algorithm of jEPlus+EA, EnergyPlus simulation determines the annual energy used for district heating, *H*_*S*(*y*_0_,…,*y*_14_)_, and for district cooling, *C*_*S*(*y*_0_,…,*y*_14_)_, in the room model. Pareto front is a useful concept when solving optimisation problems with multiple objective functions. A solution *x* of an optimisation problem with objective functions *C*_1_, *C*_2_, … is a Pareto solution if the values *C*_1_(*x*), *C*_2_(*x*), … are not simultaneously dominated by any other solution. In our setting, a shading variant *S*(*y*_0_,…,*y*_14_) is a Pareto solution if no other shading variant $S(y_{0}^{\prime },\ldots ,y_{14}^{\prime })$ satisfies both $H_{S(y_{0}^{\prime },\ldots ,y_{14}^{\prime })}<H_{S(y_{0},\ldots ,y_{14})}$ and $C_{S(y_{0}^{\prime },\ldots ,y_{14}^{\prime })}<C_{S(y_{0},\ldots ,y_{14})}$.

The Pareto front, which is a set of all Pareto solutions, is usually found by the simple cull algorithm [52]. It starts with an empty set *PF* and proceeds iteratively through all generated solutions. Each such solution *x* is compared to each solution *p* already in *PF*: if *p* dominates *x*, then *x* is discarded, while if *x* dominates *p*, then *p* cannot be a Pareto solution, so *p* is removed from *PF*. Otherwise, if *x* is incomparable to all solutions in *PF*, then *x* is added to *PF* as well. At the end of this iterative process, which takes quadratic time in worst case, the set *PF* will contain the Pareto front.

Since jEPlus+EA was instructed to perform optimisation for two objective functions, it had to update the Pareto front with each new generation, and its size was gradually increasing with the number of generations. Due to quadratic worst case time of the simple cull algorithm, after several hundred generations jEPlus+EA was spending significantly more time (6–7 minutes) updating the Pareto front and preparing the next generation than actually running EnergyPlus to simulate it (about 30 seconds). We were stopping genetic optimisation then, but not before it went through at least 400 generations. Since this was 3–4 times more than the number of generations needed for a population to become mature, one could expect that genetic optimisation was able to come near the true Pareto front by then. As a side note, representative approximation of Pareto front in the presence of increasing proportion of nondominated solutions, especially for optimization problems with many objectives, represents current research topic in the field of evolutionary optimization—for examples of different ways to deal with this problem the reader is referred to [23, 53, 54] and the references contained therein.

District heating and cooling values *H*_*S*_ and *C*_*S*_ for a shading variant *S* represent different types of end energy obtained with different equipment. They can be combined into a single value using equipment efficiency and source energy conversion factors, which represent the amount of source energy needed to produce a unit of end energy. Let *e*_*H*_ and *f*_*H*_ be, respectively, the efficiency and the source energy conversion factor for district heating, and similarly let *e*_*C*_ and *f*_*C*_ be the efficiency and the source energy conversion factor for district cooling. Total equivalent source energy *ESE*_*S*_ needed for district heating and cooling may then be found as

$$\begin{array}{c} ESE_{S}=\frac{f_{H}}{e_{H}}H_{S}+\frac{f_{C}}{e_{C}}C_{S}. \end{array}$$ (1)

While current values of efficiencies and source energy conversion factors may be found in literature [55], they are susceptible to change over time and may have different values at other locations (and in other countries). Hence it can be of interest to know which among the Pareto solutions found may have optimal value of *ESE* for different values of *f*_*H*_, *e*_*H*_, *f*_*C*_ and *e*_*C*_. An important observation here is that *f*_*H*_, *e*_*H*_, *f*_*C*_ and *e*_*C*_ are independent of a shading variant *S*, so that for any particular choice of their values total equivalent source energy *ESE*_*S*_ may be considered as just a linear combination of *H*_*S*_ and *C*_*S*_ with constant coefficients. As such, the set of shadings with the same value of *ESE* represents a line with the slope $-\frac{1}{r}$ for the ratio

$$\begin{array}{c} r=\frac{f_{C}}{e_{C}}/\frac{f_{H}}{e_{H}} \end{array}$$

of efficiencies and source energy factors, provided that *H*_*S*_ values are given on *x*-axis and *C*_*S*_ on *y*-axis. The shading variant *S* with the minimum value of *ESE*_*S*_ then belongs to the leftmost of these lines, so that all other shading variants are found to the right of this line. As already observed by one of the authors in [56], this means that such shading variant necessarily belongs to the convex hull of the Pareto front, defined as the smallest convex polygon that contains all Pareto solutions, which can be obtained by the Fortune’s variant of the Graham’s scan method [28]. The convex hull is usually determined by a much smaller number of Pareto solutions (dozens instead of hundreds in current optimisation studies), which facilitates their analysis and discussion. Moreover, each Pareto solution that is a vertex of the convex hull belongs to two of its sides, say *a* and *b*, and it represents a Pareto solution with the optimal value of *ESE* for all choices *f*_*H*_, *e*_*H*_, *f*_*C*_ and *e*_*C*_ for which the slope $-\frac{1}{r}$ falls between the slopes of sides *a* and *b*.

## Results and discussion

### Similarity of Pareto fronts of general and simplified optimisation studies

**Fig 2:**
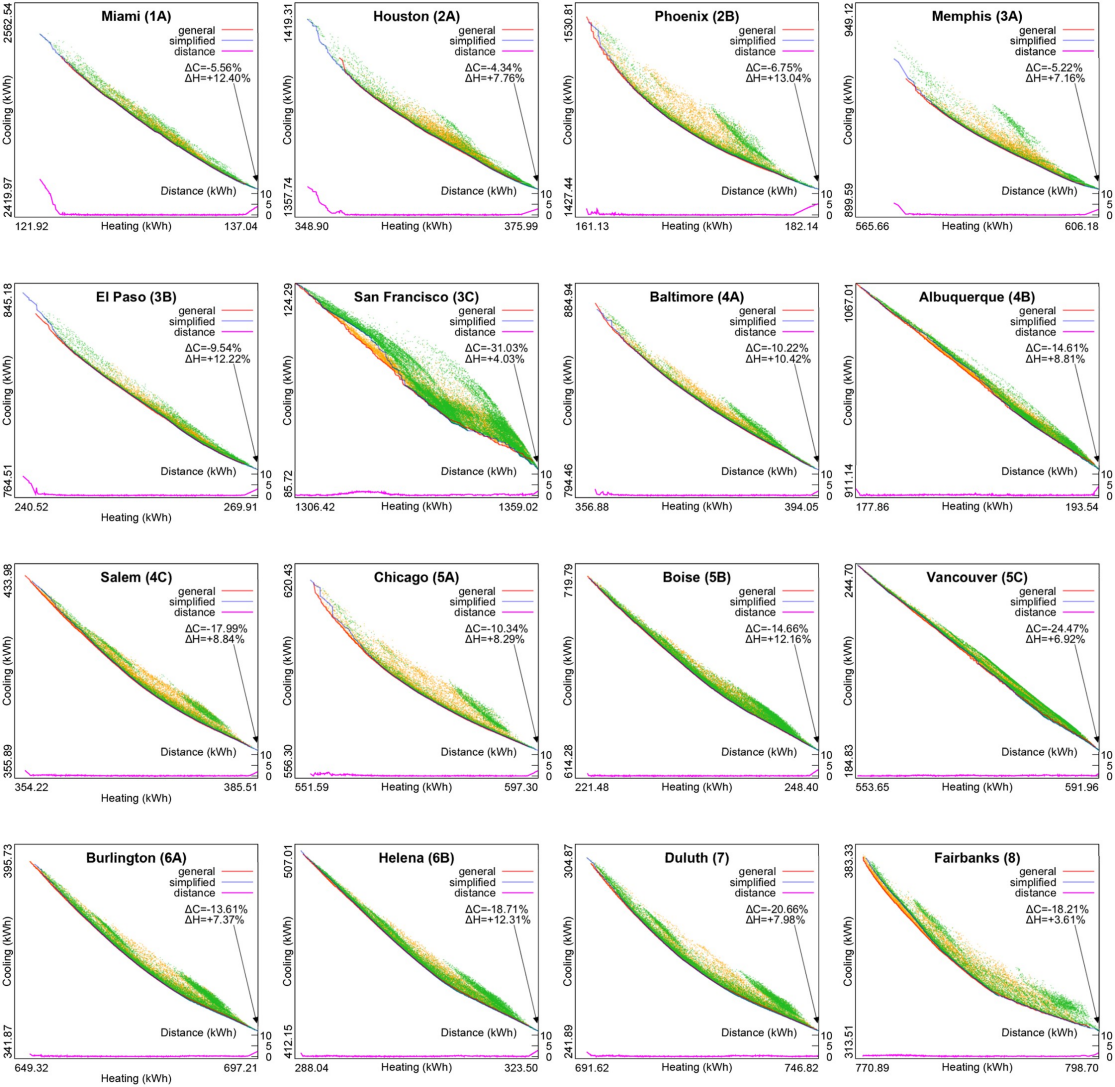
Pareto fronts for both general and simplified optimisation studies. Yellow dots represent all shading variants simulated in the general optimisation study, while green dots represent all shading variants simulated in the simplified optimisation study. Minimum heating demand is for the base case of the model without shading, and Δ*H* represents the relative increase of the maximum heating demand among obtained Pareto solutions with respect to the base case. Similarly, maximum cooling demand is for the base case of the model without shading, and Δ*C* represents the relative decrease of the minimum cooling demand among obtained Pareto solutions with respect to the base case. Graph at the bottom of each diagram represents Euclidean distance from each Pareto solution in one Pareto front to the closest Pareto solution in the other Pareto front. As the upper bound set on figure resolution may prevent distinguishing details of these diagrams, their enlarged versions are added in S1 File for easier viewing.

Table 2 presents basic results from both optimisation studies, while Fig 2 visualises obtained Pareto fronts for all locations. The upper left corner of each diagram in this figure corresponds to the base case of apartment room model without shading that necessarily represents a Pareto solution and a vertex of its convex hull, as it has the lowest heating demand and the highest cooling demand among all shading variants due to unobstructed solar heat gain. The base case without shading is a Pareto solution with extremal value (equal to zero) for each shading parameter and the genetic algorithm managed to reach it only for Albuquerque and San Francisco in the general optimisation study. Note that diagrams in Fig 2 have different axis scale factors for easier visualisations of Pareto fronts that are extended toward the lower right corner. This way it becomes easier to see that the Pareto fronts for the general optimisation study, in which the depths of NURBS control points were set independently, and for the simplified optimisation study, in which the NURBS control points were divided into six groups with the same depth set within each group, are indeed very similar. To quantify this similarity, we calculated the root mean square distance (*RMSD*) of each Pareto solution to the other Pareto front, as well as the maximum distance found between a Pareto solution and the other Pareto front, which are shown in Table 2. For this, the distance of a Pareto solution *P* from the other Pareto front *f* is defined as the smallest Euclidean distance between *P* and any Pareto solution that belongs to *f*, with heating and cooling demands taken as their coordinates.

**Table 2:** Basic results from general and simplified optimisation studies.

Location	General optimisation study			Simplified optimisation study			*RMSD* (kWh)	*MAXD* (kWh)
	# gen.	Pareto	Convex hull	# gen.	Pareto	Convex hull		
Miami	452	883	23	498	948	26	1.398	16.800
Houston	425	916	24	453	937	30	1.201	13.292
Phoenix	489	832	32	517	884	30	0.567	5.287
Memphis	484	841	27	442	943	40	0.250	5.606
El Paso	471	913	28	451	911	25	0.463	9.101
San Francisco	570	572	5	562	940	5	0.713	2.118
Baltimore	440	949	25	500	985	30	0.184	3.014
Albuquerque	509	860	13	570	783	12	0.475	4.549
Salem	627	1057	35	472	934	27	0.221	2.616
Chicago	466	941	33	439	927	26	0.266	2.494
Boise	484	973	21	455	1073	28	0.270	3.006
Vancouver	504	795	10	462	1246	9	0.220	1.109
Burlington	470	877	29	454	953	28	0.250	2.466
Helena	473	863	23	444	1035	22	0.306	2.893
Duluth	490	799	28	451	990	41	0.218	2.140
Fairbanks	471	991	29	612	796	27	0.365	1.747

The *# gen*. column shows for how many generations genetic algorithm was run with jEPlus+EA. The *Pareto* and *Convex hull* columns give the numbers of shading variants in the Pareto front and its convex hull for each optimisation study. The *RMSD* column represents the root mean square distance between a Pareto solution of each optimisation study and the nearest point on the Pareto front of the other optimisation study. The *MAXD* column represents the maximum of these distances.

Note that higher values of the maximum distance in the cases of Miami, Houston, Memphis and El Paso actually represent distances between the upper left corners of their Pareto fronts. In each of these cases, simplified optimisation study was able to come closer to the base case without shading than general optimisation study whose control point depths were set in the lower resolution, 0.25m steps. The case of San Francisco is also instructive: although the maximum distance between its Pareto fronts is not large compared to other locations due to different axis scale factors used in diagrams in Fig 2, apparent discrepancy in shapes of its Pareto fronts is easily recognisable from a high ratio between the root mean square distance and the maximum distance in this case.

Overall, small values of root mean square distance from Table 2 show that Pareto solutions with restricted control point structure may replace Pareto solutions with independently set control points with, on average, negligible differences in heating and cooling demands. This confirms our hypothesis that in studies of external window shading in high-rise apartment buildings it is sufficient to use simpler NURBS lined shadings obtained by partitioning control points into six groups such that all control points in the same group have equal depths, with groups corresponding to lower part of the western fin, upper part of the western fin, joint of the western fin and the overhang, internal part of the overhang, joint of the overhang and the eastern fin and the remaining part of the eastern fin.

### Convex hulls of the Pareto fronts for the simplified optimisation study

**Fig 3:**
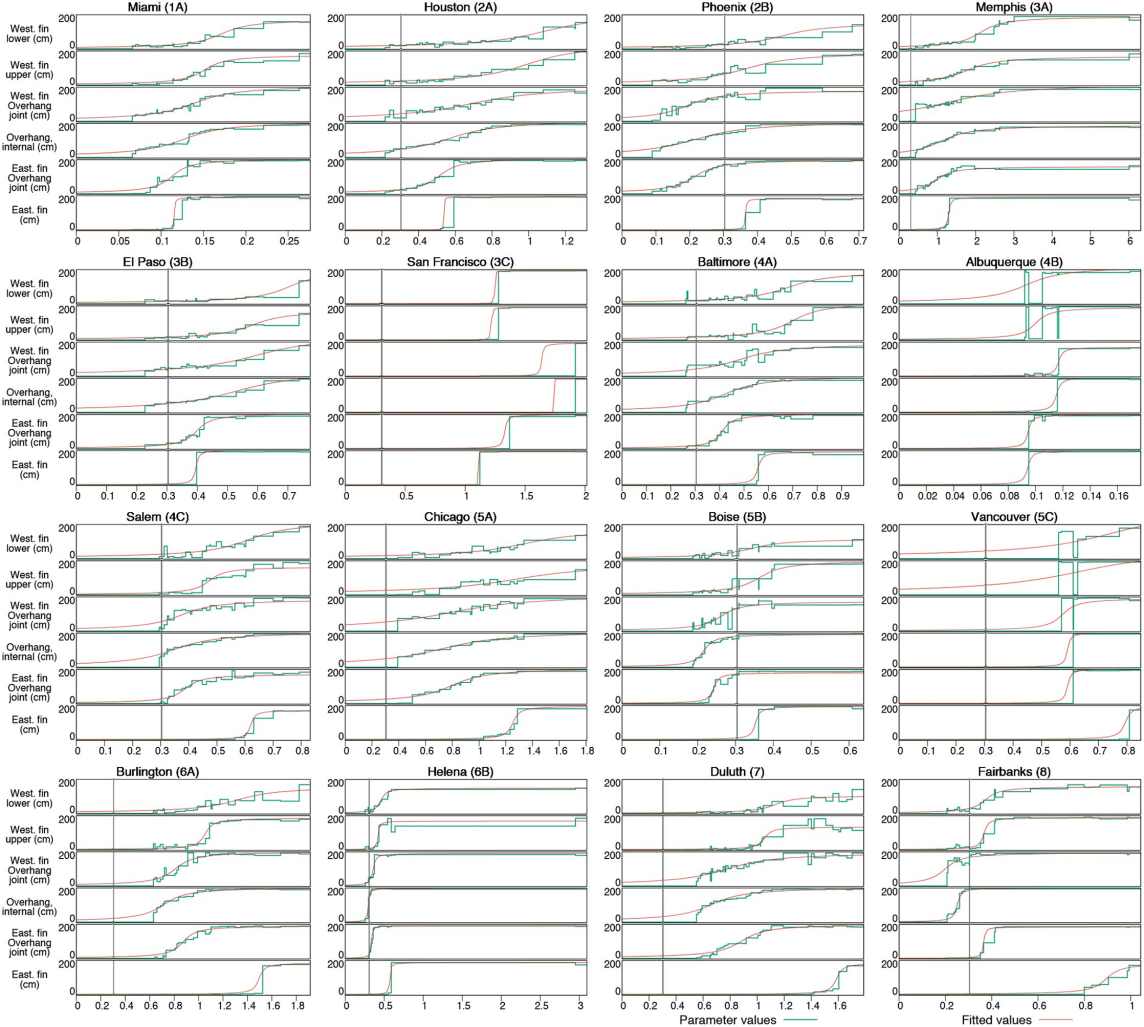
Shading parameters of solutions in the convex hull of Pareto fronts for simplified optimisation study. Horizontal axis shows the ratio $\frac{f_{C}}{e_{C}}/\frac{f_{H}}{e_{H}}$ of efficiencies and source energy conversion factors for district cooling and heating. Vertical axis depicts depth values for the corresponding group of control points in the shading. Green horizontal steps determine parameter values: *y*_0_ = *y*_1_ for the lower part of the western fin, *y*_2_ = *y*_3_ for the upper part of the western fin, *y*_4_ for the joint of the western fin and the overhang, *y*_5_ = ⋯ = *y*_9_ for the internal part of the overhang, *y*_10_ for the joint of the overhang and the eastern fin, and *y*_11_ = ⋯ = *y*_14_ for the remaining part of the eastern fin. The steps are stretched between the values of ratios $\frac{f_{C}}{e_{C}}/\frac{f_{H}}{e_{H}}$ for which the corresponding shading in the convex hull has the minimum total equivalent source energy demand. Gray vertical line is placed at *r* = 0.3038, which corresponds to current efficiencies and source energy conversion factors for district cooling and heating in USA [55]. Red line shows fitted arctangent function for depths of each control point group.

While the number of Pareto solutions in both studies reaches above a thousand for some locations, the number of shadings forming the convex hull of the Pareto front is substantially smaller, in the order of 20–40, making their analysis more tractable. Each shading in the convex hull, whose control point depths are visualised in Fig 3, has an associated range of values for the ratio $r=\frac{f_{C}}{e_{c}}/\frac{f_{H}}{e_{H}}$ of efficiencies and source energy conversion factors for district cooling and heating, for which it minimises total equivalent source energy for district heating and cooling demands. It can be seen from Eq (1) that *r* represents relative importance of the cooling demand with respect to the heating demand, since for fixed value of *f*_*H*_/*e*_*H*_ total equivalent source energy *ESE*_*S*_ of each shading *S* is directly proportional to the linear combination *H*_*S*_ + *rC*_*S*_. Thus for sufficiently small values of *r* window shading is not needed as the reduction of cooling demand that it yields during summer is overcome by the increase in heating demand during winter. On the other hand, for sufficiently large values of *r* reduction in cooling demand becomes more important so that optimal shadings get increasing depths, almost always to the full extent allowed here.

**Fig 4:**
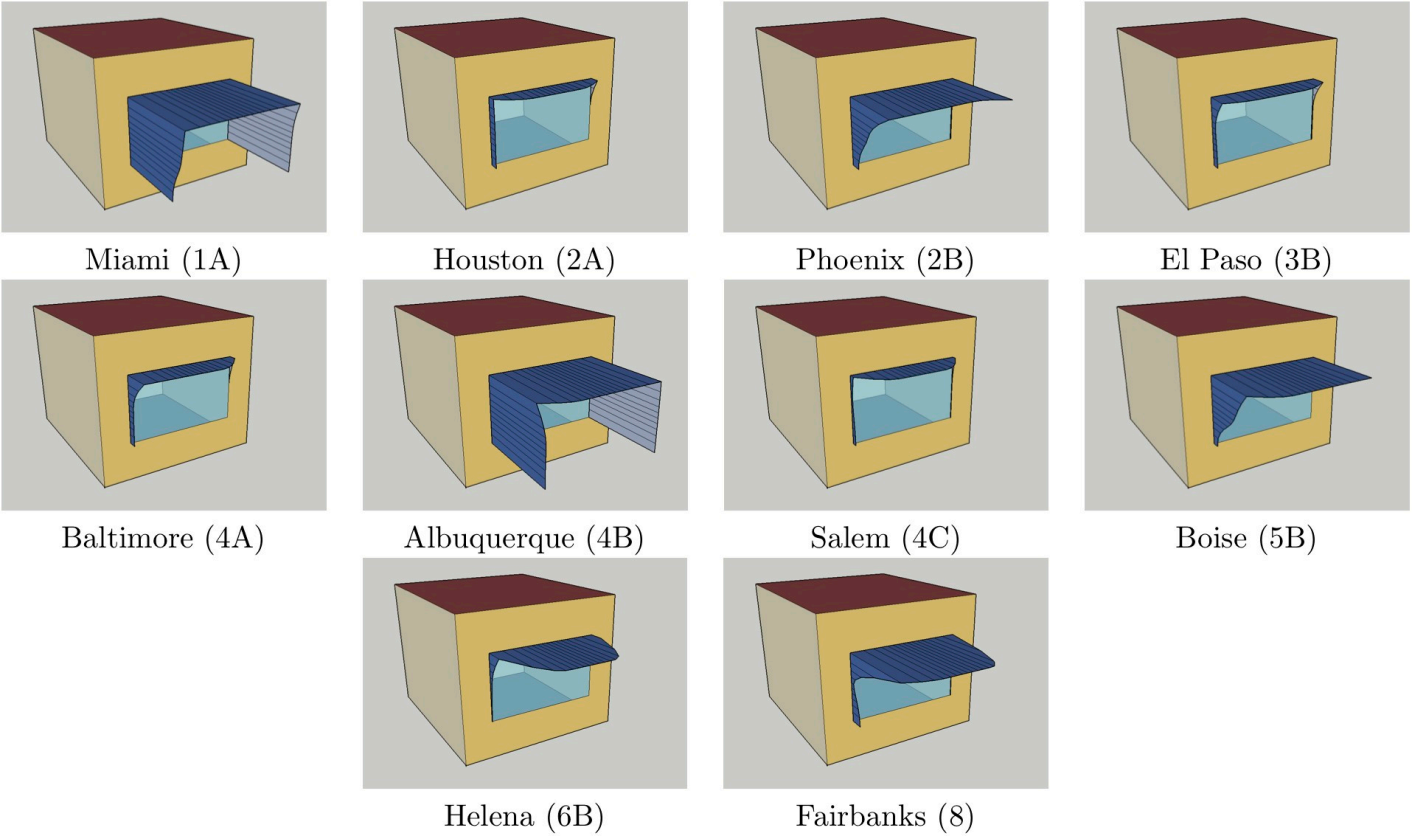
Sketchup visualisations of convex hull shadings with minimal equivalent source energy for *r* = 0.3038. In the remaining six locations: Memphis, San Francisco, Chicago, Vancouver, Burlington and Duluth, minimal equivalent source energy is attained in the base case of the model without any shading.

According to [55], district heating in USA locations has efficiency *e*_*H*_ = 0.3 and uses natural gas with source energy conversion factor *f*_*H*_ = 1.092, while district cooling has efficiency *e*_*C*_ = 3.0 and uses electricity with source energy conversion factor *f*_*C*_ = 3.317, so that the value of the ratio *r* is approximately 0.3038. While these values are subject to change with development of energy technology, one can expect that *r* would change relatively little in the foreseeable future. Parameters of the convex hull shadings that minimise equivalent source energy for *r* = 0.3038 are listed in Table 3, while their visualisations are shown in Fig 4. The minimal value of *r*, min_*r*_, for which depths of convex hull shadings start to become positive, is larger than 0.3038 in a number of locations: Memphis (min_*r*_ = 0.427), San Francisco (min_*r*_ = 1.122), Chicago (min_*r*_ = 0.392), Vancouver (min_*r*_ = 0.560), Burlington (min_*r*_ = 0.630) and Duluth (min_*r*_ = 0.551), so that the optimal choice in these locations is the base case of a room model without any shading. Moderately sized convex hull shadings of depth at most 0.66m are optimal in Houston, El Paso, Baltimore and Salem. These shadings have noticeable overhang that prevents solar heat gain at midday during summer, together with a subtle western fin that apparently manages to regulate solar heat gain by the afternoon sun. The next group of optimal convex hull shadings, those for Phoenix, Boise, Helena and Fairbanks, have a very pronounced overhang. While it may be certainly welcome during Phoenix’s hot summers, it is unlikely it is needed in Fairbanks. Rather, this means that some shading of window in the model is necessary for Fairbanks as well, but due to its northern location the overhang has to be extended further outside in order to prevent even a small amount of midday solar heat gain in summer. In such cases it is better to use a shading device in the vertical plane of the window or a better performing solar control glazing. Finally, for Miami and Albuquerque the spread of cooling demands of Pareto solutions is much higher than the spread of their heating demands, making their Pareto fronts very steep (note again that diagrams in Fig 2 use different axis scale factors). As a consequence, whenever *r* ≥ 0.263 for Miami and *r* ≥ 0.169 in Albuquerque the optimal convex hull shading becomes the one with the smallest cooling demand and the largest heating demand found during genetic optimisation, which produces an almost total enclosure of the model window thanks to large depths of its control point groups. These extremely deep shadings show that optimization process is trying to prevent diffuse radiation from reaching the window as well, as it apparently has large impact on cooling load in these climates. In addition to a vertically placed shading device or a better performing solar control glazing, a smaller window-to-wall ratio is another adequate alternative in these locations.

**Table 3:** Convex hull shadings with minimal equivalent source energy for *r* = 0.3038, together with best shadings found in a separate genetic optimisation with equivalent source energy as its single objective.

Location	Shading from	*y*_0_ = *y*_1_ (cm)	*y*_2_ = *y*_3_ (cm)	*y*_4_ (cm)	*y*_5_ = … = *y*_9_ (cm)	*y*_10_ (cm)	*y*_11_ = … = *y*_14_ (cm)	*ESE*_*S*_ (kWh)
Miami	convex hull	159	189	197	195	199	187	3174.51
	single-objective	198	176	199	184	183	200	3174.60
Houston	convex hull	19	21	20	52	21	0	2833.91
	single-objective	8	21	30	55	17	0	2833.70
Phoenix	convex hull	24	72	143	144	175	2	2234.66
	single-objective	33	44	132	136	151	20	2235.33
Memphis	convex hull	0	0	0	0	0	0	3108.43
	single-objective	9	7	1	21	7	6	3113.96
El Paso	convex hull	16	13	46	59	32	2	1802.86
	single-objective	9	15	45	50	25	8	1803.59
San Francisco	convex hull	0	0	0	0	0	0	4892.79
	single-objective	0	8	0	0	1	0	4894.21
Baltimore	convex hull	17	19	66	66	16	0	2273.57
	single-objective	17	20	13	92	24	0	2275.34
Albuquerque	convex hull	194	197	169	197	198	199	1711.89
	single-objective	182	198	196	195	198	170	1712.62
Salem	convex hull	11	5	13	66	9	1	1768.54
	single-objective	10	15	7	69	3	0	1768.05
Chicago	convex hull	0	0	0	0	0	0	2693.77
	single-objective	10	0	0	18	0	8	2701.37
Boise	convex hull	15	95	132	169	170	0	1578.54
	single-objective	35	82	153	158	191	0	1578.53
Vancouver	convex hull	0	0	0	0	0	0	2285.84
	single-objective	1	2	2	12	1	3	2291.11
Burlington	convex hull	0	0	0	0	0	0	2801.08
	single-objective	0	1	0	7	0	0	2801.87
Helena	convex hull	2	8	41	154	32	0	1607.09
	single-objective	6	43	6	122	17	7	1608.74
Duluth	convex hull	0	0	0	0	0	0	2854.57
	single-objective	16	24	2	28	25	0	2865.64
Fairbanks	convex hull	30	5	179	199	2	1	3221.63
	single-objective	15	17	169	197	10	7	3223.23

In order to confirm that the convex hull shadings listed in Table 3, found in genetic optimisation whose objectives were both heating demand *H*_*S*_ and cooling demand *C*_*S*_, also optimise equivalent source energy *ESE*_*S*_ = 3.640*H*_*S*_ + 1.10567*C*_*S*_, obtained for current values of *f*_*H*_, *e*_*H*_, *f*_*C*_ and *e*_*C*_, we performed additional genetic optimisation with *ESE*_*S*_ as its single objective using the same genetic parameters: population of 16 simplified shadings, crossover rate 0.8, mutation rate 0.0041, 500 generations. Best shadings found in this single-objective optimisation are listed in the second row for each location in Table 3. For each location these two shadings are similar to each other, with occassional increased depth of one control point group usually compensated by decreased depth of a neighboring control point group. Quite evident from this table is the overall agreement between equivalent source energy of the optimal convex hull shadings and the optimal shadings from a single-objective optimisation, with differences larger than 0.103% only for locations in which the optimal convex hull solution is the base case of the model without shading, a candidate solution with extremal depths of control point groups (all zeros) that is hard to reach by genetic optimisation. As a matter of fact, the convex hull solutions are often performing slightly better than the optimal shadings from a single-objective optimisation, verifying usefulness of the convex hull of Pareto front, which with its relatively small cardinality gives optimal solutions for all convex linear combinations of objectives.

### Fitting the convex hull of Pareto fronts for the simplified optimisation study

An interesting observation from Fig 3 is that most diagrams in it have a similar, sigmoid-like shape: once it becomes positive, depth of almost every control point group seems to increase more-or-less linearly with the increase in *r*, until it reaches its maximum value. This linear trend is, of course, not perfect. A possible reason for rugged appearance of depth change diagrams is that the influences of depths of six control point groups on heating and cooling demands are not mutually independent, so that genetic algorithm may have several directions available to get closer to the Pareto front with each new generation. As genetic algorithm was instructed to optimise heating and cooling demands only, without paying attention to other properties of shadings, this may have led it to a quite different distribution of depths from other aesthetically more pleasing shadings with similar heating and cooling demands.

Nevertheless, shape of depth diagrams in Fig 3 suggests that they could be approximated by a sigmoid function. Out of five sigmoids that we had tried (logistic, hyperbolic tangent, arctangent, inverse square root unit and softsign function), best approximations were obtained with the arctangent function

$$\begin{array}{c} f\left(r\right)=L(\frac{1}{2}+\frac{\arctan(kr-m)}{\pi }), \end{array}$$

where *L* is the maximum value of *f*, *k* determines the slope at the point of inflection (i.e., at the middle point) and $\frac{m}{k}$ gives the middle point. Control point group depth data were fitted by the Levenberg-Marquardt algorithm [29, 30] using gnuplot’s fit function with starting values *L* = 200, *k* = 30 and *m* = 5. Obtained fitting functions are shown in Fig 3, while their parameters and root mean square deviations are given in Table 4.

**Table 4:** Parameters of fitting functions for depths of control point groups of shadings in the convex hull of Pareto fronts for simplified optimisation study.

Control point group	Value	MIA	HOU	PHO	MEM	ELP	SF	BAL	ALB	SAL	CHI	BOI	VAN	BUR	HEL	DUL	FAI
Western fin, lower part	*L*	176.55	210.49	157.09	194.90	203.38	198.17	195.83	225.69	224.66	178.61	118.76	305.81	161.68	152.35	106.72	162.84
	*k*	38.80	4.00	10.04	2.19	11.58	447.57	7.76	48.48	8.48	2.91	14.54	4.49	3.86	19.50	8.86	21.91
	*m*	6.63	4.35	4.78	4.66	8.16	556.96	5.28	4.52	5.19	3.90	4.95	3.36	5.09	8.88	10.05	8.15
	*RMSD*	10.79	11.35	11.12	9.83	7.24	0.12	18.57	69.43	22.38	14.73	12.22	63.36	22.79	11.39	16.51	12.82
Western fin, lower part	*L*	181.06	249.79	197.83	174.86	178.39	195.72	228.50	197.90	168.10	188.43	206.95	278.26	189.71	171.87	137.18	192.95
	*k*	51.94	4.50	8.98	1.64	11.52	238.92	10.42	125.96	23.43	2.17	19.63	4.00	20.93	69.16	24.31	73.92
	*m*	7.87	4.47	3.30	2.91	6.72	291.38	7.28	12.58	11.09	2.77	7.14	2.53	22.07	29.13	25.61	27.07
	*RMSD*	11.99	11.07	17.68	10.55	9.95	0.71	20.50	81.41	21.17	18.95	23.29	88.45	13.48	19.52	17.67	8.70
Joint of overhang and western fin	*L*	216.53	211.53	187.00	227.87	249.02	197.01	198.78	173.46	190.72	222.03	178.18	197.87	205.66	195.25	201.44	200.64
	*k*	26.76	3.31	13.46	0.74	5.96	96.56	7.02	458.45	10.62	2.13	25.09	25.19	8.55	39.94	3.71	18.40
	*m*	3.73	2.27	2.59	0.96	3.56	158.25	3.38	53.59	3.97	1.68	6.58	14.44	6.94	14.20	2.87	3.64
	*RMSD*	11.87	15.77	16.87	16.80	10.68	0.61	22.93	33.64	22.66	16.68	27.75	75.61	18.00	17.80	16.92	19.16
Overhang, internal part	*L*	218.44	227.19	218.35	194.98	254.53	199.00	219.64	213.92	212.70	220.12	199.13	208.51	209.34	202.29	210.97	205.68
	*k*	26.55	4.81	8.19	1.69	5.91	4E+12	8.78	402.42	8.60	2.70	49.64	135.14	6.99	96.57	5.22	83.36
	*m*	3.37	2.72	1.79	1.87	3.13	7E+12	3.71	46.43	3.00	1.94	10.73	79.85	5.00	28.43	3.46	20.92
	*RMSD*	14.97	9.69	11.10	9.04	8.57	3.9E-11	11.08	43.76	10.08	9.51	12.63	13.57	10.22	7.82	9.69	12.59
Joint of overhang and eastern fin	*L*	210.41	214.10	204.63	163.56	211.48	203.58	207.34	202.84	175.82	214.45	184.39	208.41	195.73	195.10	205.99	188.22
	*k*	61.09	12.25	19.29	3.10	26.98	66.38	30.72	536.10	22.85	5.06	98.60	117.86	12.40	96.95	7.32	192.56
	*m*	6.92	6.29	4.15	3.00	10.75	88.31	12.62	50.67	8.85	4.03	23.87	69.69	10.82	32.52	6.47	70.29
	*RMSD*	19.62	11.89	8.52	11.28	19.39	13.10	11.21	28.76	19.22	10.96	17.47	14.94	15.29	10.39	15.23	5.61
Eastern fin	*L*	193.64	196.75	186.44	196.23	208.26	198.55	198.64	207.80	176.41	201.82	199.61	207.73	190.67	191.23	191.19	188.76
	*k*	1355.51	741.47	478.67	26.45	143.01	3617.00	76.92	477.68	89.37	27.48	124.37	60.65	30.87	105.49	28.71	20.86
	*m*	156.53	398.87	174.53	34.29	56.58	4017.44	42.86	45.05	55.45	34.45	43.89	48.16	46.13	60.19	45.57	18.64
	*RMSD*	4.96	1.56	1.48	15.13	27.70	0.62	21.76	35.92	3.07	11.12	13.86	19.59	10.47	10.20	5.67	8.08

For each control point group, the first three rows give the parameters of the fitting function $f\left(r\right)=L(\frac{1}{2}+\frac{1}{\pi }\arctan\left(kr-m\right))$. *RMSD* is the root mean square deviation of fitted values from the actual control point groups’ depths, given in centimeters. Location abbreviations: MIA = Miami, HOU = Houston, PHO = Phoenix, MEM = Memphis, ELP = El Paso, SF = San Francisco, BAL = Baltimore, ALB = Albuquerque, SAL = Salem, CHI = Chicago, BOI = Boise, VAN = Vancouver, BUR = Burlington, HEL = Helena, DUL = Duluth, FAI = Fairbanks.

One should observe here that there is a number of cases in which the optimal depth of control point group experiences a sudden jump from the minimum to the maximum value, greatly increasing the slope of successive data points. This results in very large values of the slope parameter *k* during fitting, which should be taken with caution as subtle changes in optimal depth values, that may arise from another run of the genetic algorithm, might change *k* substantially. The extreme example is certainly San Francisco: thanks to unusually concave part of its Pareto front in both its upper left half and the lower right quarter (see Fig 2), it has just five solutions in the convex hull. This yields sudden jumps of control point group depths in Fig 3, especially for the internal part of the overhang where optimal depth is 0cm for *r* < 1.92 and 199cm for *r* ≥ 1.92, resulting in *k* approximately equal to 3.78 ⋅ 10^12^. Similar sudden jumps, resulting with *k* in the order of several hundreds, are present in diagrams of depths of the eastern fin control point group in majority of locations. A related feature to which the fitting algorithm is also sensitive appears for lower and upper parts of the western fin in Albuquerque and Vancouver, as well as for joint of the western fin and the overhang in Vancouver: optimal depths in these cases suddenly rise from almost minimal to almost maximal values, and then quickly drop to almost minimal value before rising again later to the maximum value. In order to accommodate these temporary drops, the fitting algorithm substantially decreases the value of *k* losing much of its approximation quality, as is evident from high root mean square deviation values in these cases in Table 4. Both of these anomalies are actually related to the appearance of long sides in the convex hull of Pareto fronts: in cases when genetic algorithm manages to find two particularly successful, but distant Pareto solutions *s* and *t*, the line joining their heating and cooling demands will be below all other Pareto solutions between *s* and *t*, so that none of them will be selected in the convex hull. While Pareto solutions close to each other in heating and cooling demands usually have small differences between depths of same control point groups, distant Pareto solutions *s* and *t* are expected to have significantly different values for several of their parameters. These anomalies might perhaps be overcome if genetic algorithm could be instructed to produce a more convex Pareto front by favouring solutions belonging to the current convex hull while selecting parents to produce offsprings for the next generation.

Without the outliers mentioned above: all control point groups for San Francisco, lower and upper parts of the western fin for Albuquerque and lower part, upper part and joint of the western fin with the overhang in Vancouver; the root mean square deviation *ρ* between optimal and fitted depths has average value of 14.81cm and standard deviation of 7.16cm over the remaining 85 control point groups, indicating good approximating quality of the obtained fitting functions. While this means that, on average, shadings with fitted depth values will not differ much in shape from the shadings obtained by genetic optimisation, fitted shadings have an additional quality of changing their shape smoothly with increasing values of *r*.

**Fig 5:**
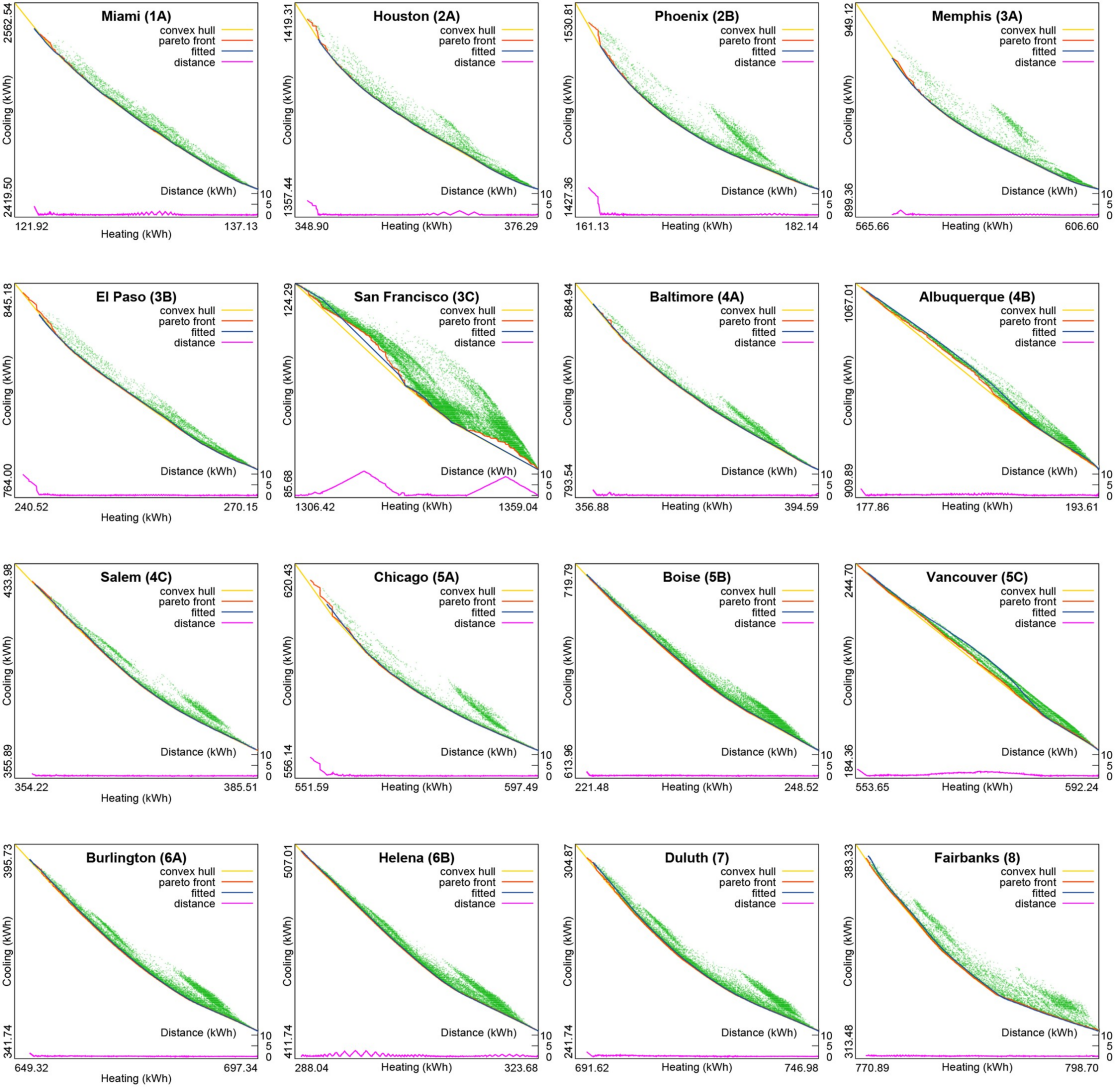
Heating and cooling demands of the Pareto solutions for the simplified optimisation study, the corresponding convex hull shadings and simplified shadings obtained by fitting control point group depths of convex hull shadings. Green dots represent all shading variants simulated in the simplified optimisation study. Graph at the bottom of each diagram represents Euclidean distance from each Pareto solution to the closest fitted shading. As the upper bound set on figure resolution may prevent distinguishing details of these diagrams, their enlarged versions are added in S2 File for easier viewing.

Next, in Fig 5 we visualise how well fitted shadings can approximate heating and cooling demands of the Pareto shadings and the convex hull shadings from the simplified optimisation study. The first thing to observe here is that, except in San Francisco, the diagram of heating and cooling demands of fitted shadings cannot reach the upper left part of the diagram for convex hull shadings, which corresponds to the base case of the model without shading. This is a consequence of the fact that even for *r* = 0 fitted depths of control point groups are larger than zero, especially for the joint of the western fin with the overhang and the internal part of the overhang (except for Albuquerque and Vancouver where the main culprit is the western fin). Further, heating and cooling demands of fitted shadings apparently diverge from the convex hull shadings in cases of San Francisco, Albuquerque and Vancouver. This is related to sudden jumps of control point group depths in San Francisco and holes in the diagrams of optimal depths of control point group related to western fin in Albuquerque and Vancouver. As explained above, these features cause large discrepancy between fitted and optimal depths for these control point groups which further implies differences in heating and cooling demands of these shadings. In the remaining locations, where the root mean square deviation between optimal convex hull depths and fitted depths is, on average, smaller than 15cm, fitted shadings turn out to approximate Pareto front rather well, with the root mean square distance between heating and cooling demands of fitted shadings and Pareto shadings at most 0.79kWh. On the other hand, the maximum Euclidean distance between heating and cooling demands of fitted shadings and Pareto shadings is at most 12.87kWh, mostly representing the distance between the upper left parts of the Pareto front and the diagram of fitted shadings for locations in which fitted control point group depths are well above zero for *r* = 0. While in colder climates heating and cooling demands of fitted shadings are, as expected, above the Pareto front, in warmer climates (Miami, Houston, Phoenix, Memphis and El Paso) heating and cooling demands of some fitted shadings actually fall below the Pareto fronts, although these improvements are marginal and localised to the upper left parts of the Pareto fronts only.

## Conclusion

In previous study [26] on optimal shading of windows in a cellular office in the PNNL office building model, where the outer edge of the shading was modeled by a NURBS curve with independently varying depths of control points, we observed that in a close vicinity of each optimal shading there exist shadings with substantially simpler structure in which control points are divided in six groups corresponding to lower part of the western fin, upper part of the western fin, joint of the western fin and the overhang, internal part of the overhang, joint of the overhang and the eastern fin, and the remaining part of the eastern fin, such that all control points within the same group have equal depth. We confirm here that the same holds for shadings of windows in a room in the PNNL high-rise apartment building model by showing that the Pareto fronts of two genetic optimisation runs, one for shadings with independently varying depths of control points and another for shadings with control points divided in groups of equal depth, have very similar shapes with the root mean square Euclidean distance between two Pareto fronts being less than 1.4kWh. This allows to use simpler NURBS lined shadings, defined by a smaller number of parameters, in future studies of external window shading in both office and apartment buildings.

We also pointed out usefulness of the convex hull of Pareto front in multiobjective optimisations, which usually contains just a fraction of the whole Pareto front. Namely, when optimisation is performed so that several objectives (such as heating and cooling demands) are simultaneously minimised, then the vertices of the convex hull represent Pareto solutions that optimise arbitrary convex linear combinations of these objectives (such as equivalent source energy). This was verified here through comparison with optimal shadings found from a separate single-objective genetic optimisation for equivalent source energy using the current values of efficiencies and source energy conversion factors for district heating and cooling.

We further showed that depths of control point groups in convex hull shadings, optimal with respect to the ratio of district heating and cooling efficiencies and source energy conversion factors, can be fitted with a particular form of the arctangent function due to their sigmoid-like shape. The obtained fitting is reasonably tight, except in cases when depths of control point groups experience sudden changes between their extremal values, a sort of anomalies that are related to the existence of long sides in the convex hull. Without such anomalies, resulting shadings with fitted control point group depths yield satisfactory approximation of heating and cooling demands for the most part of the Pareto front. This indicates that further studies might use sigmoid functions in searching for a model able to approximate optimal shadings for other building models and locations as well.

Finally, large differences are easily noticeable when one compares optimal shadings for the current ratio of district heating and cooling efficiencies and source energy conversion factors found here for a window in an apartment room model and in [26] for a window in a cellular office model. These are consequences of differences in many aspects of the underlying models, including lighting, equipment, and occupancy schedules, heating and cooling setpoints, floor area per person, lighting densities, as well as the presence of minimum lighting requirements in the cellular office model. It would be worthwhile to try in further studies to relate depths of optimal simplified shadings with such model properties and climate characteristics, in particular with incident solar radiation rates during the parts of the day that naturally correspond to the identified control point groups of shadings, in order to get closer to a comprehensive model for approximating depths of optimal simplified shadings.

## Supporting information

S1 File Enlarged versions of diagrams in Fig 2. (PDF)

S2 File Enlarged versions of diagrams in Fig 5. (PDF)
