# Supplementary figures and images for: On optimal and near-optimal shapes of external shading of windows in apartment buildings
Source: PLoS One. 2019 Feb 28;14(2):e0212710. doi: 10.1371/journal.pone.0212710 (PMC6394916; doi:10.1371/journal.pone.0212710)

# Baltimore (4A)

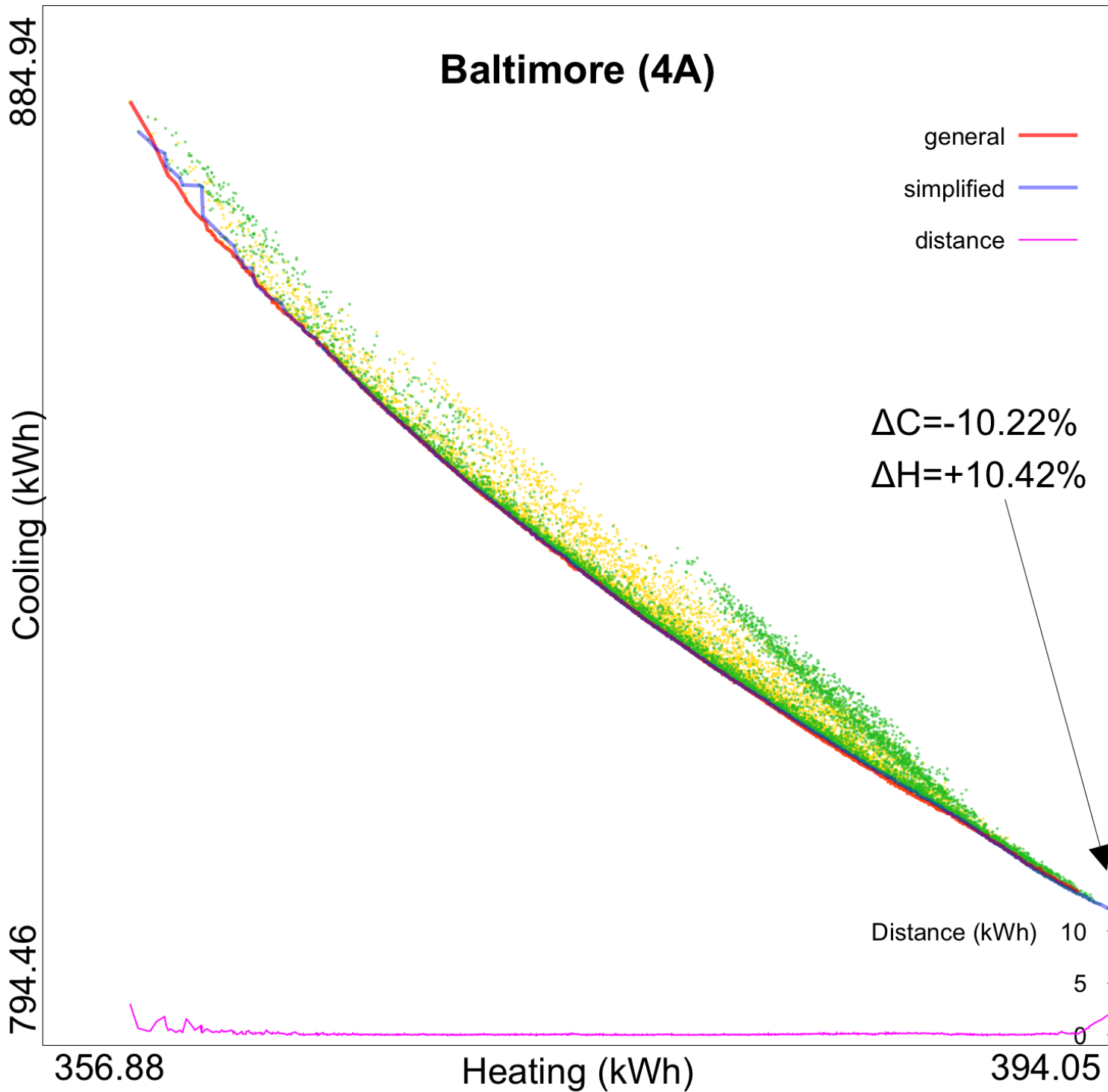

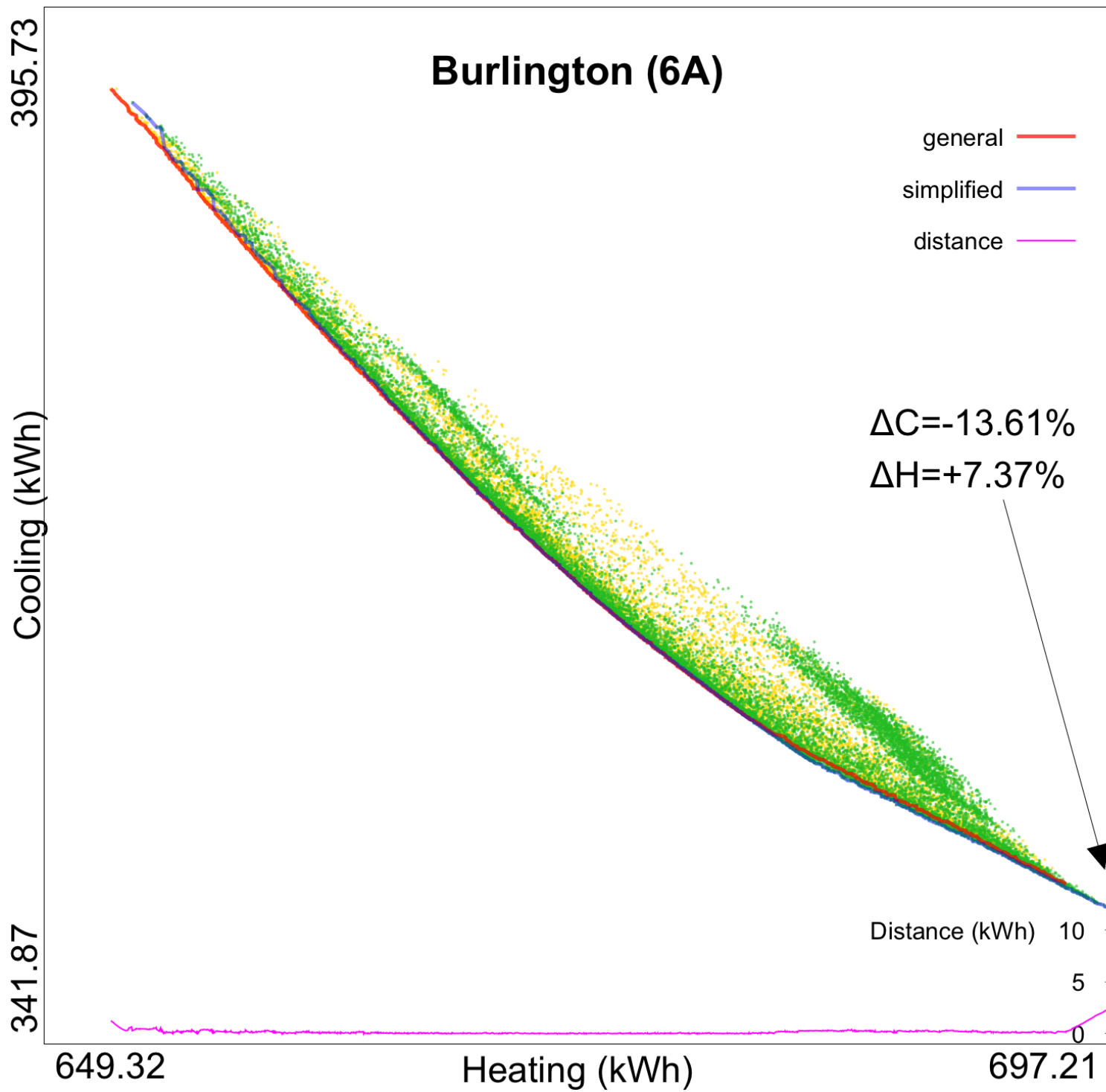

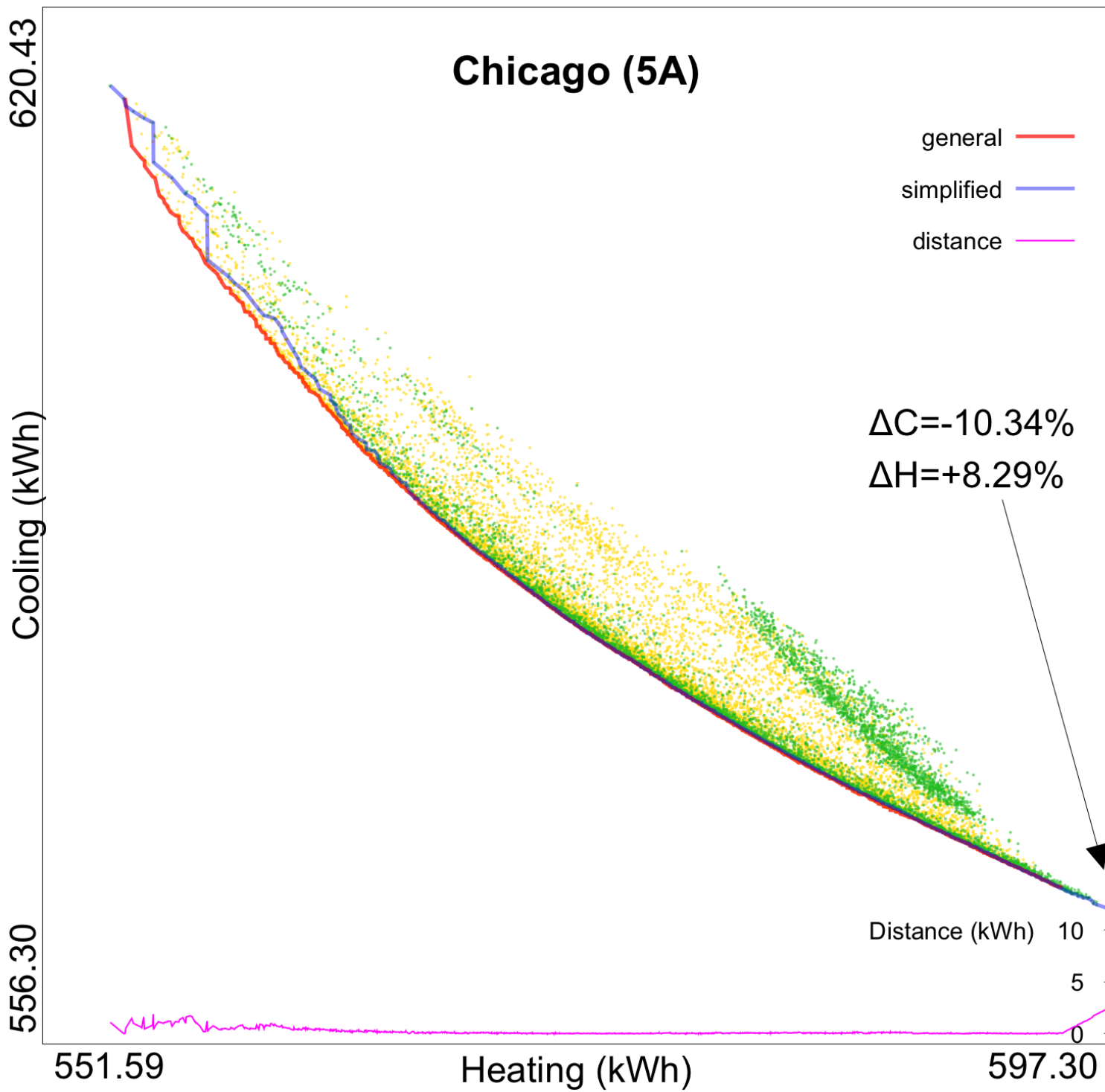

# Fairbanks (8)

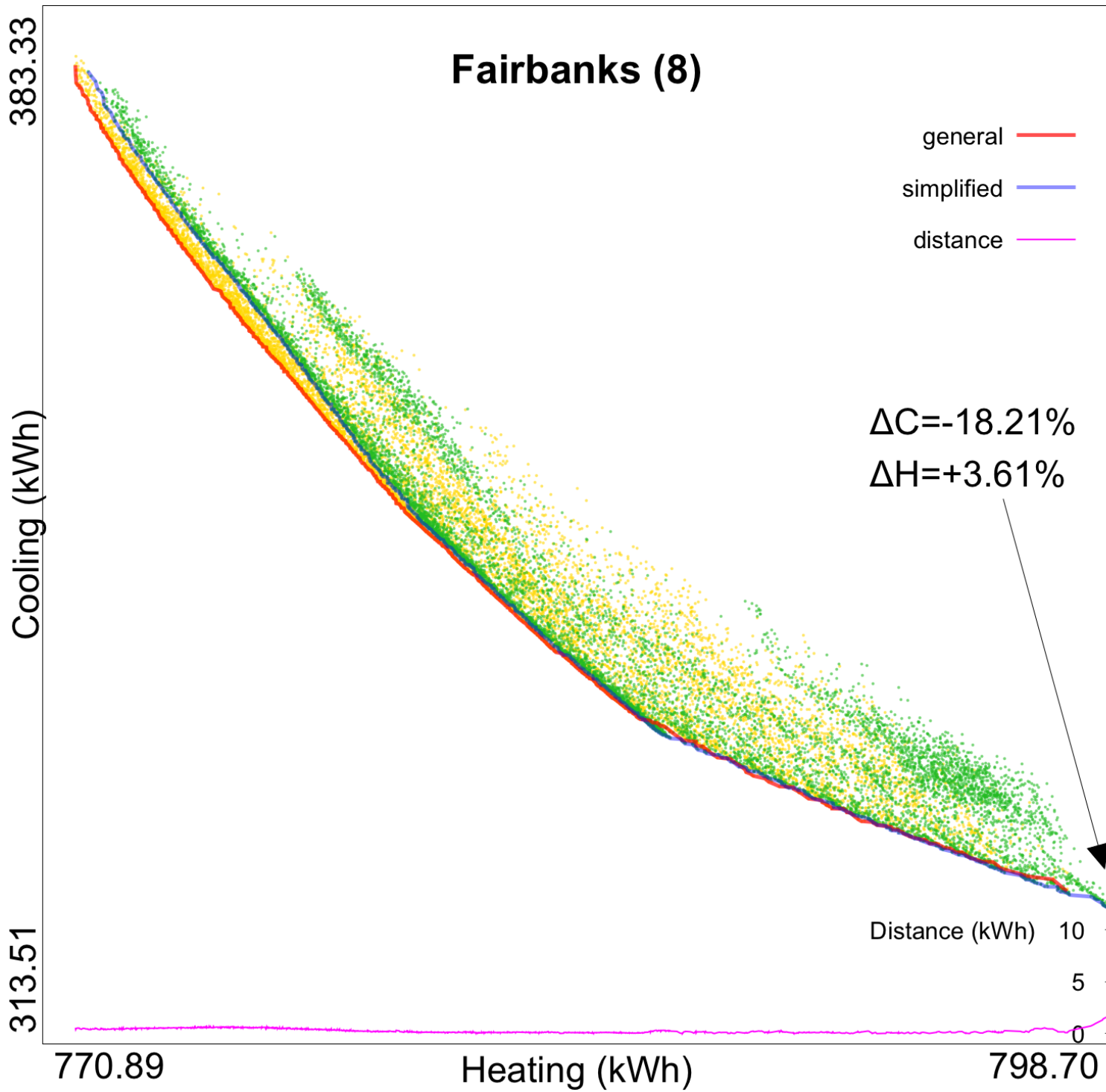

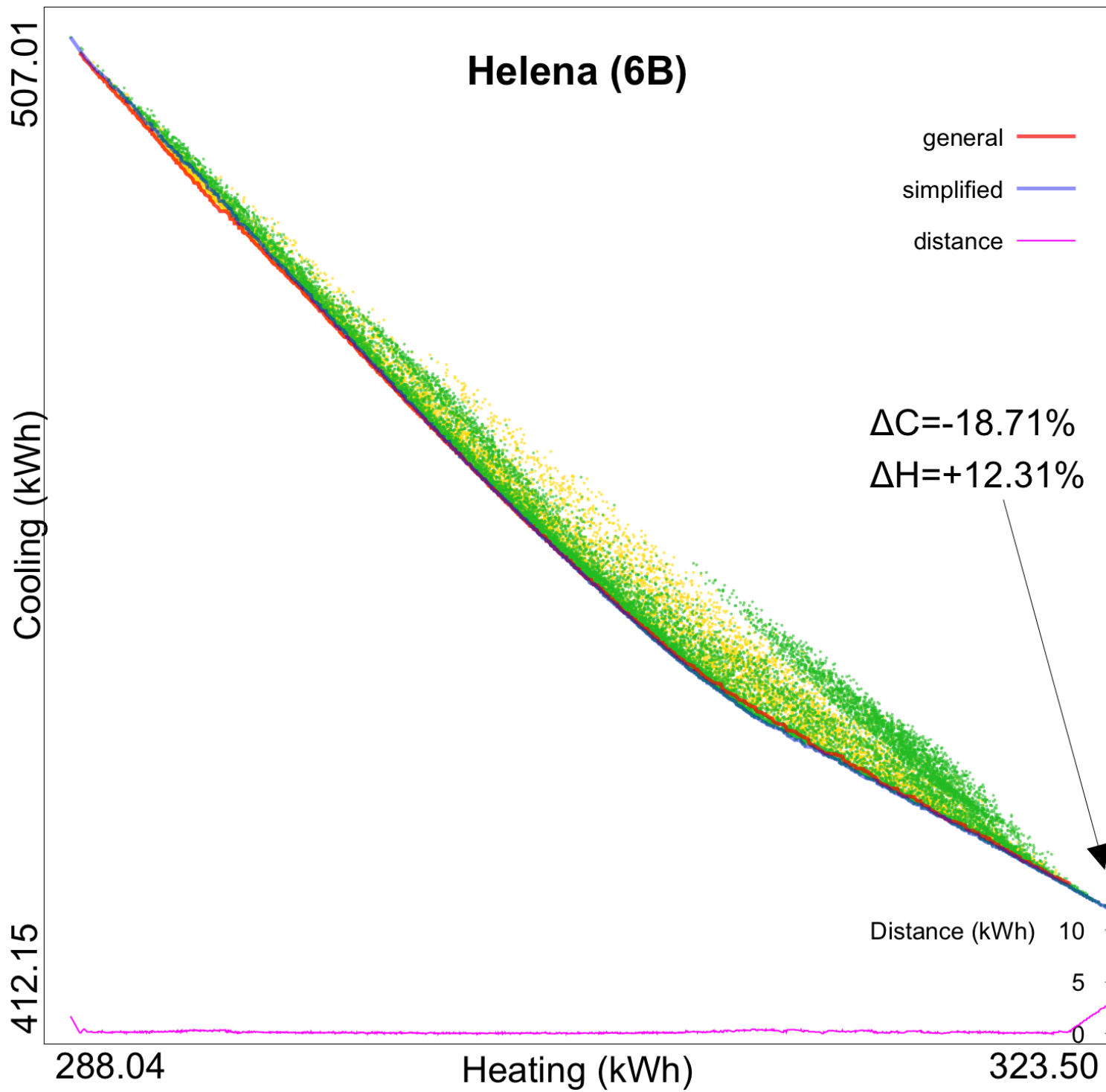

# Memphis (3A)

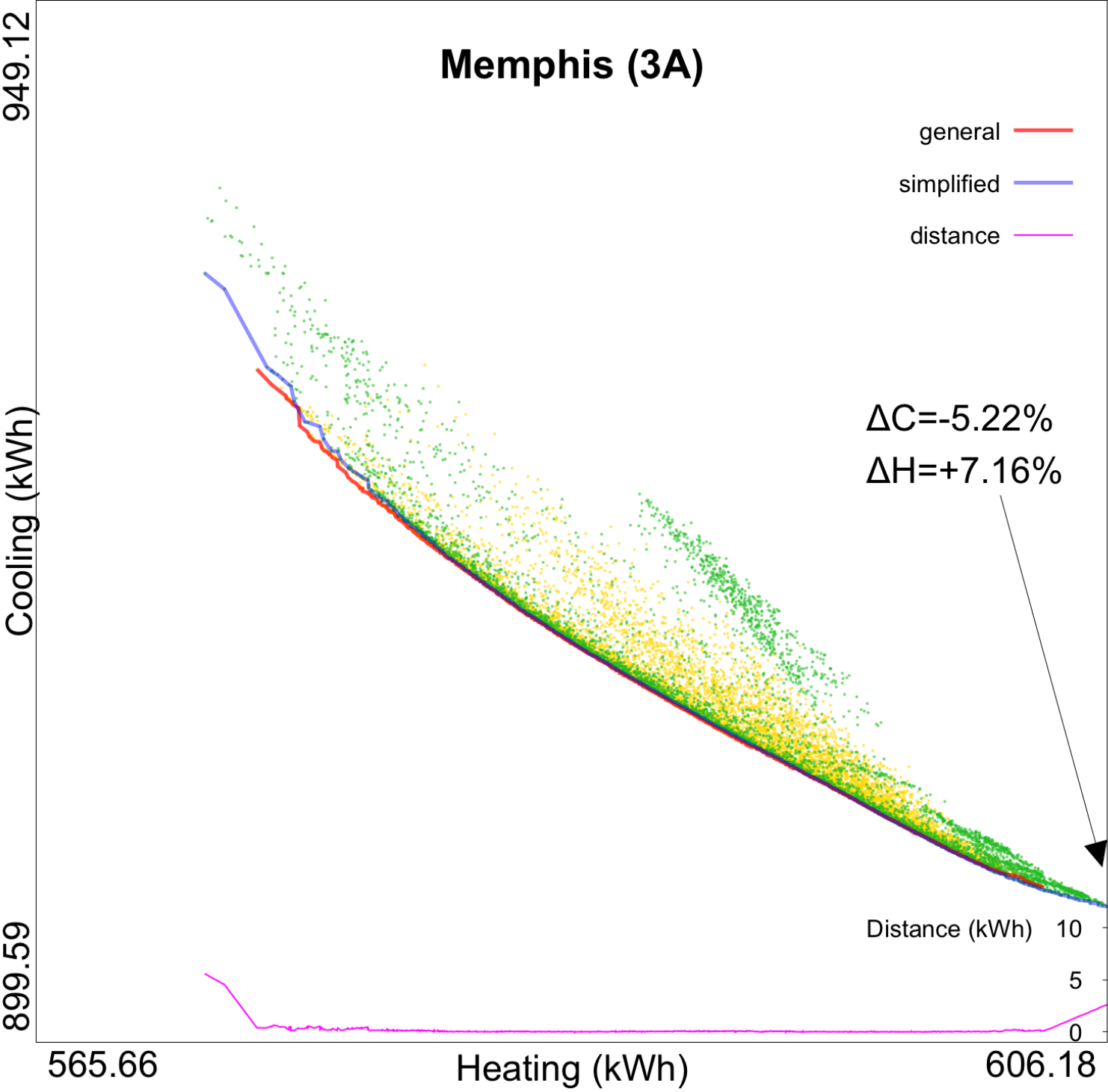

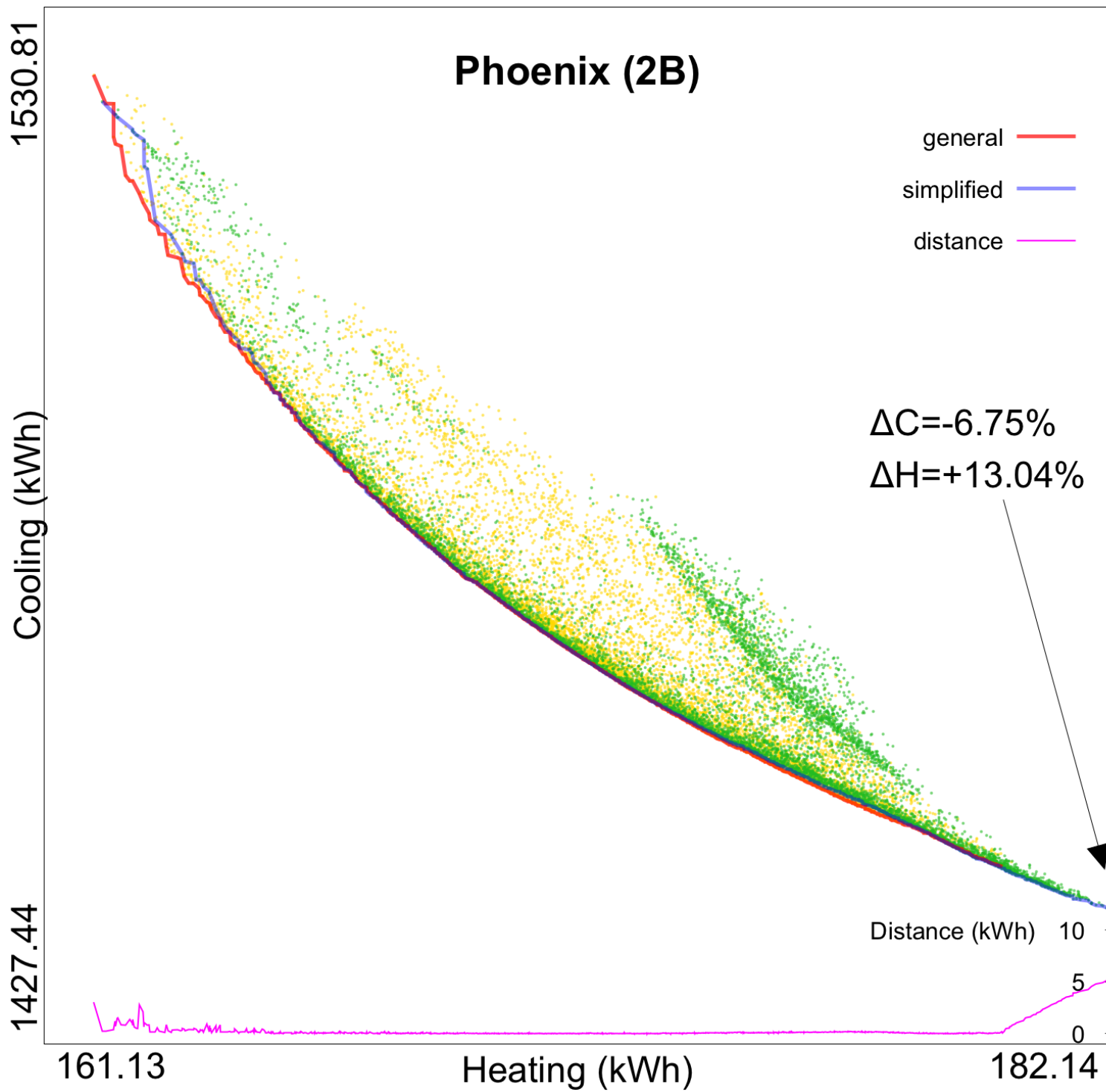

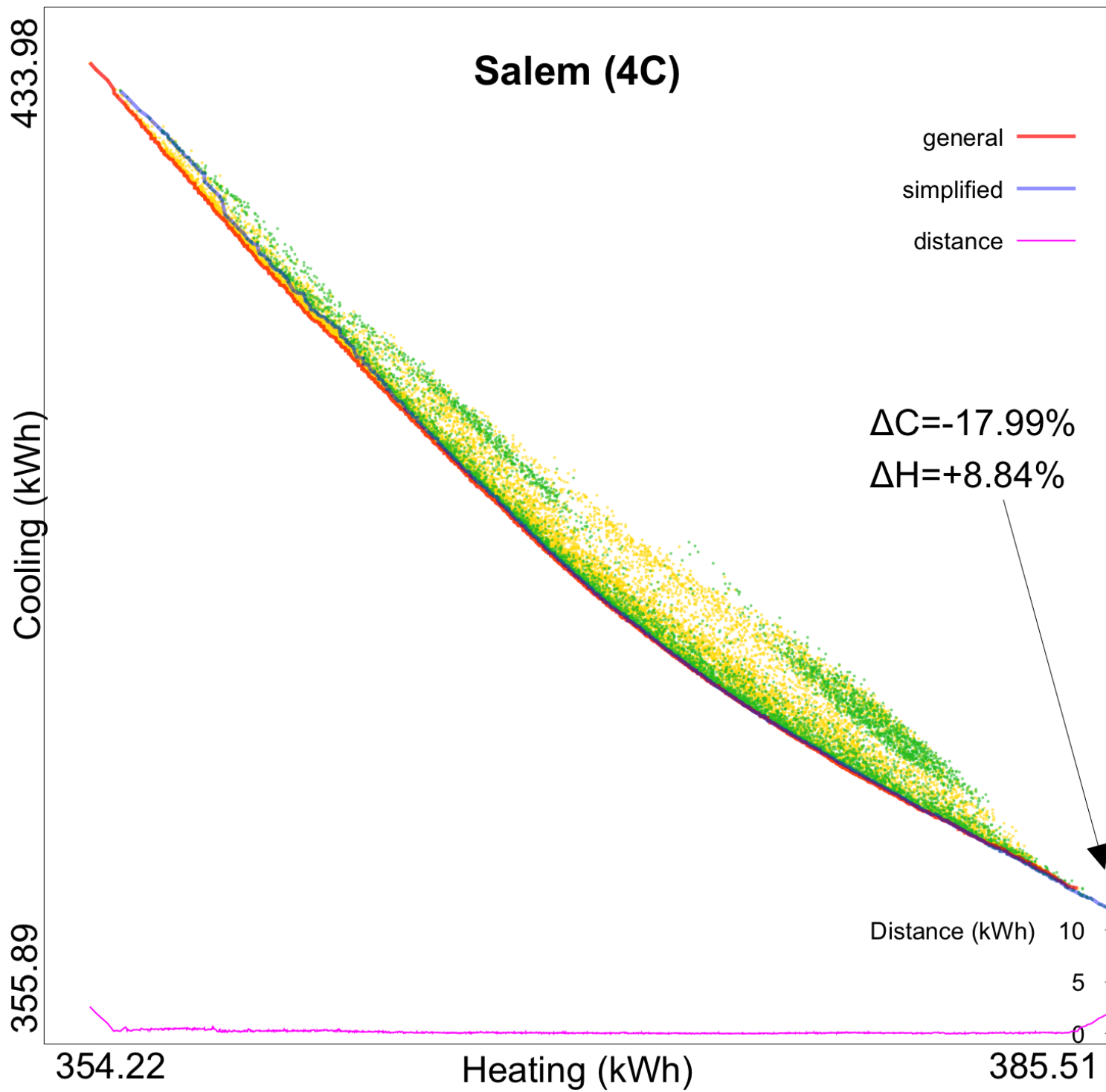

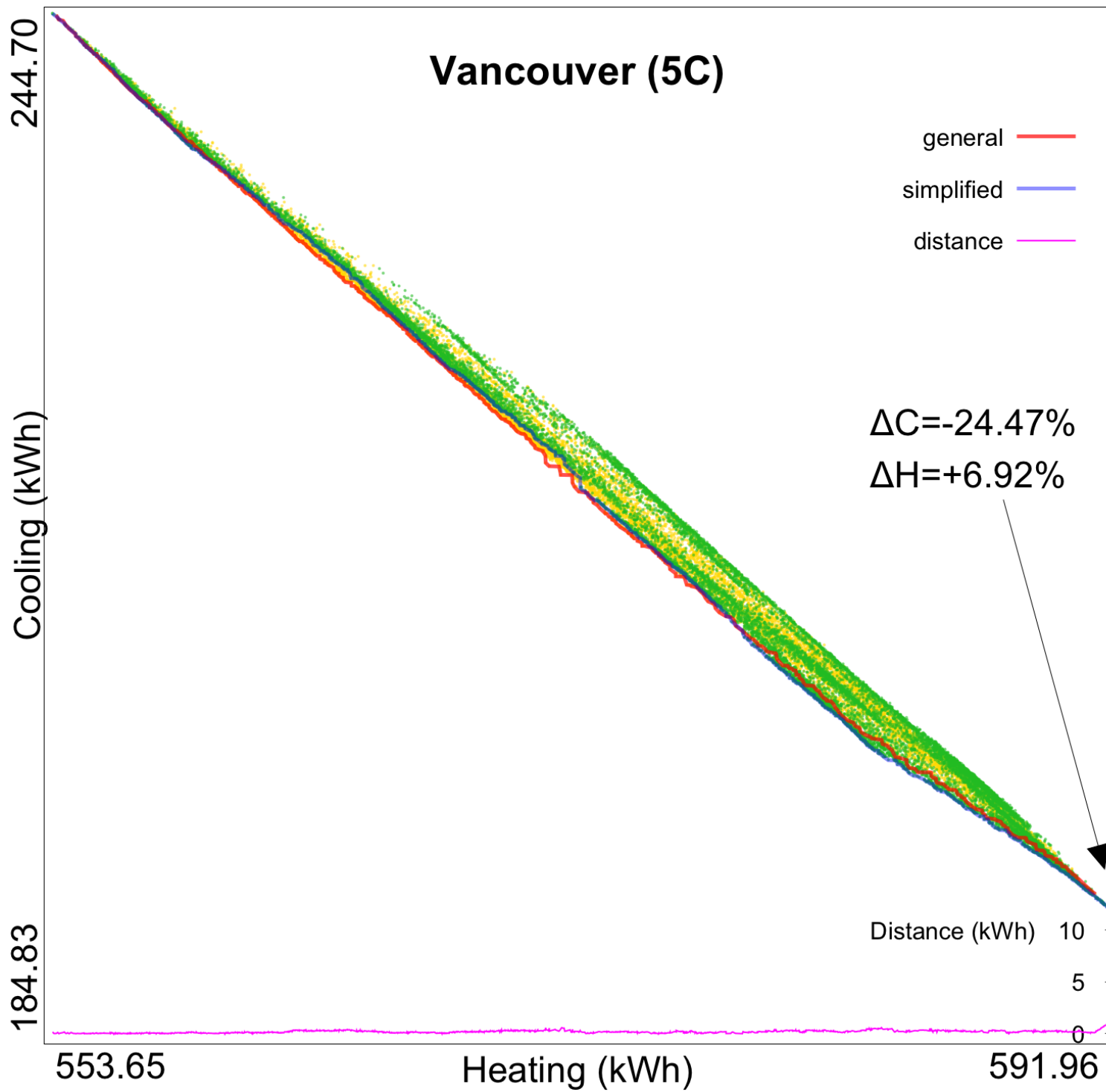

# Albuquerque (4B)

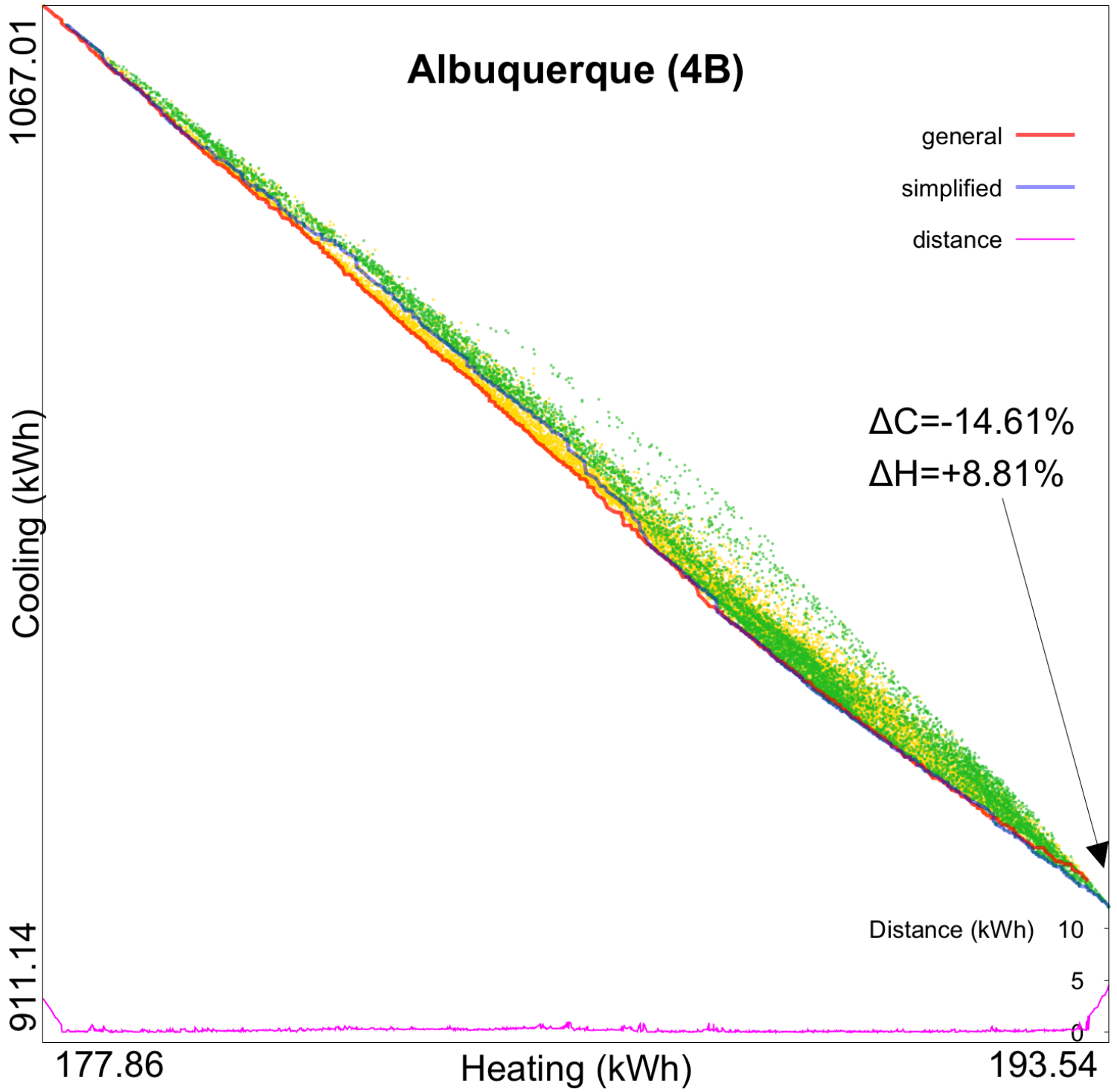

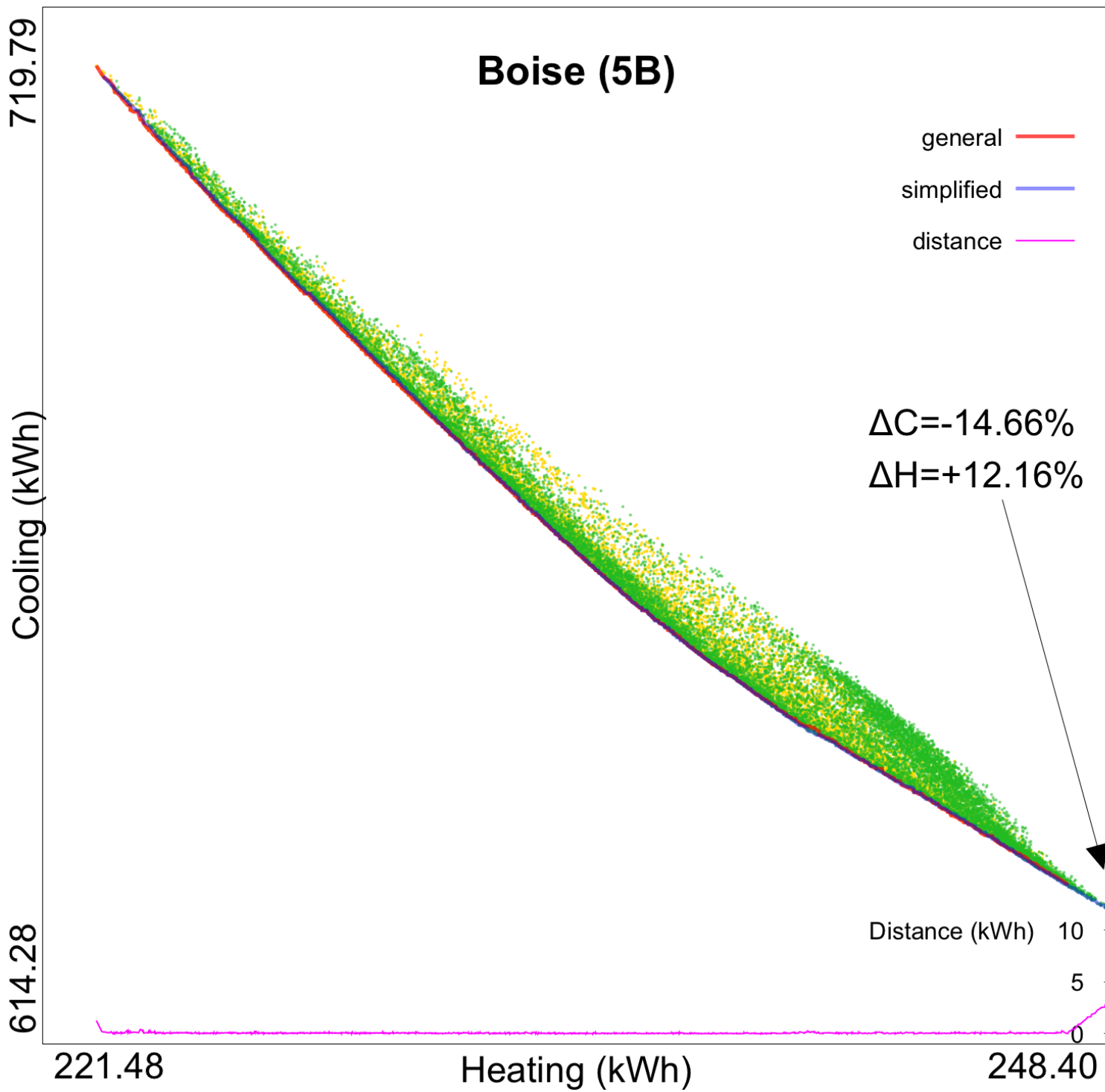

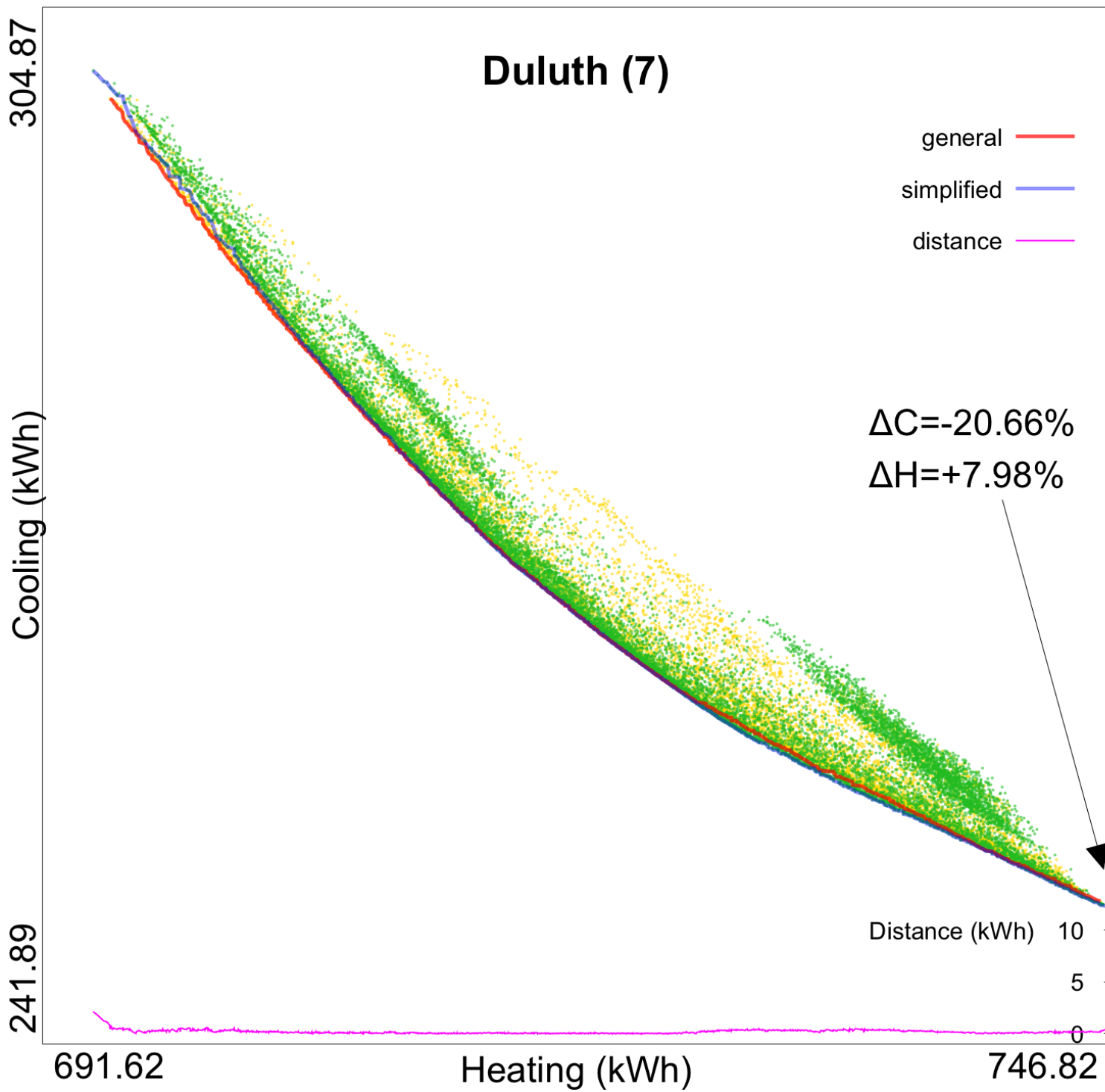

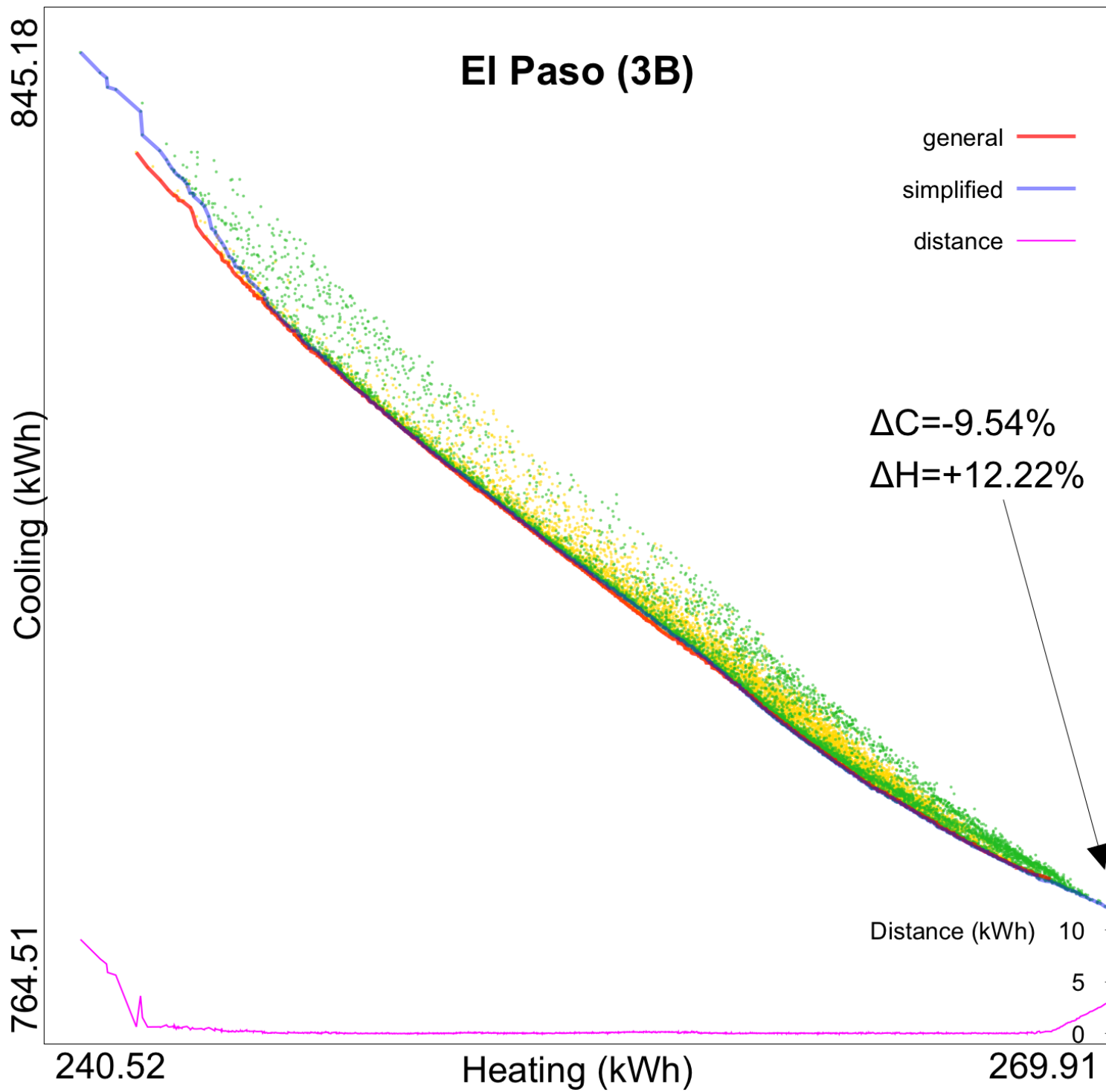

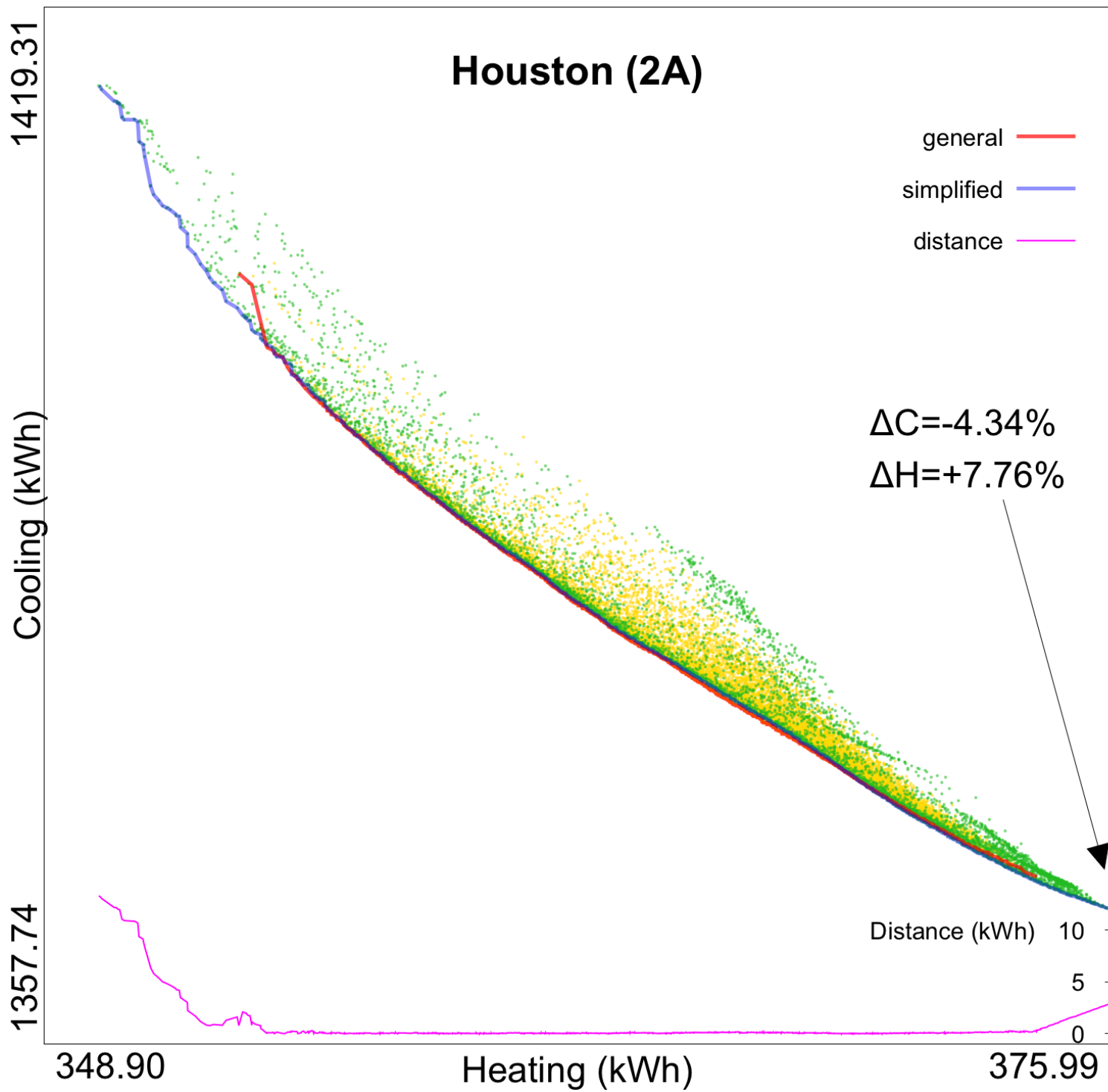

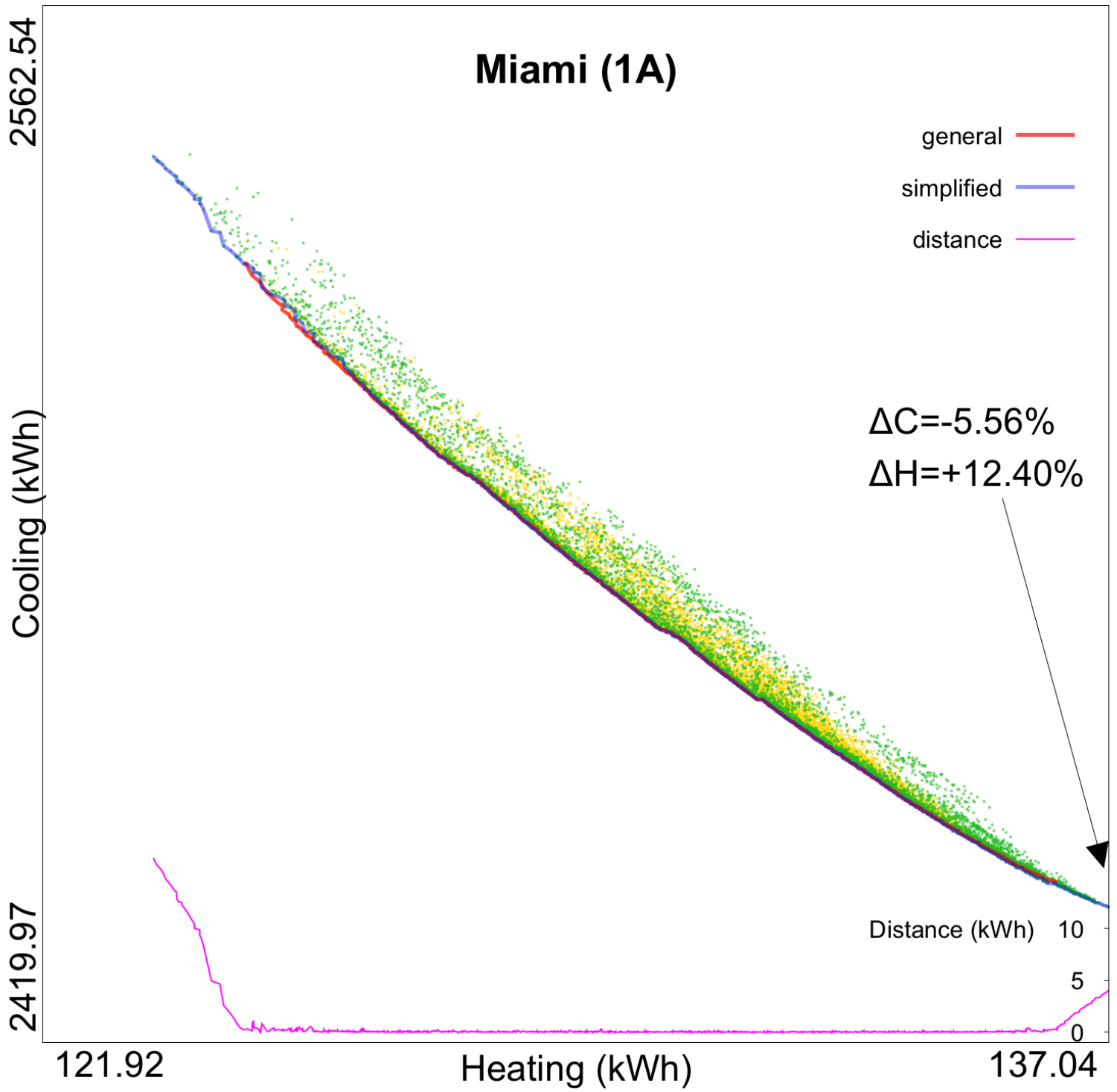

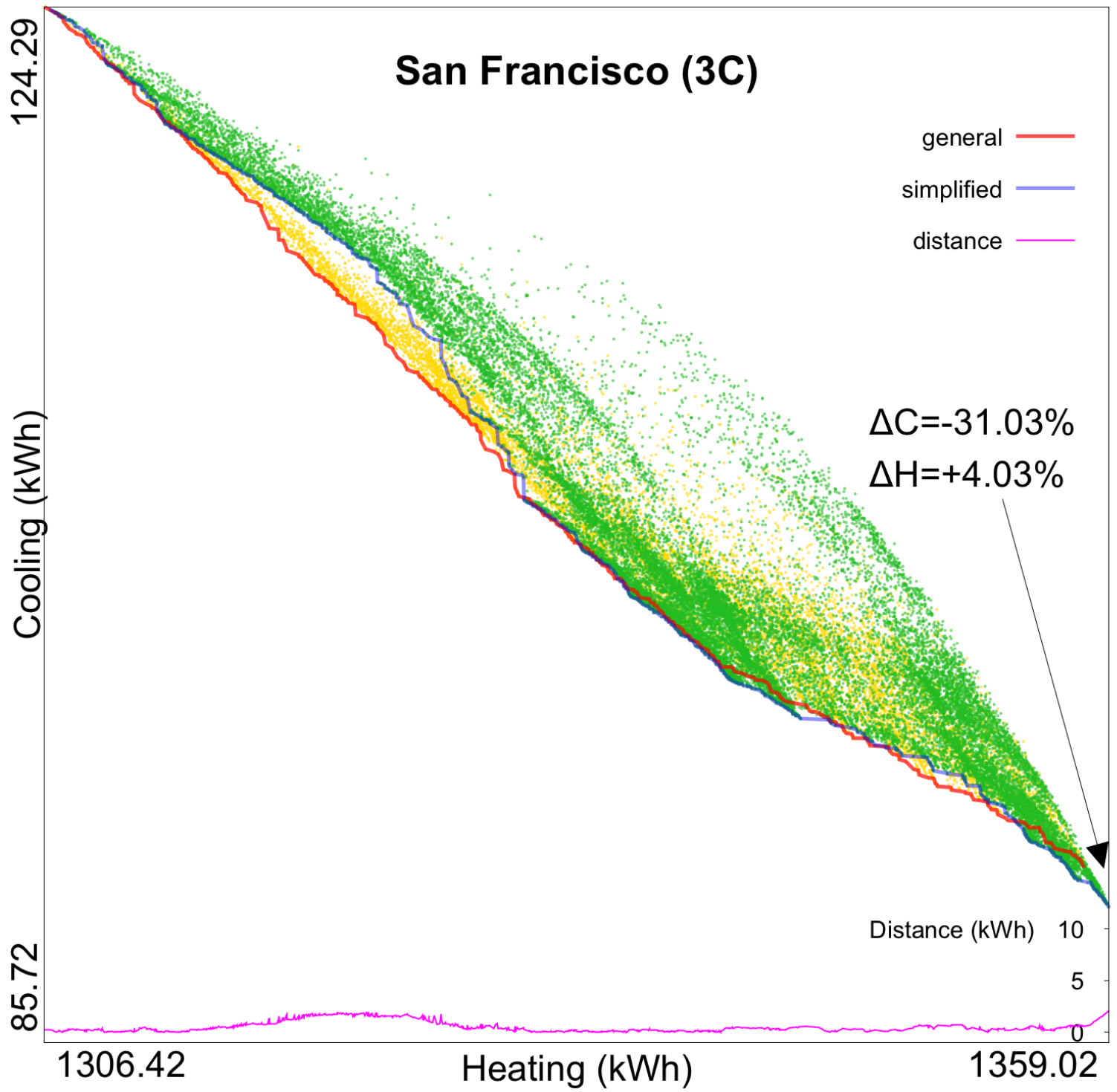

Supplement: S1 File — (PDF) [file pone.0212710.s001.pdf]

# Albuquerque (4B)

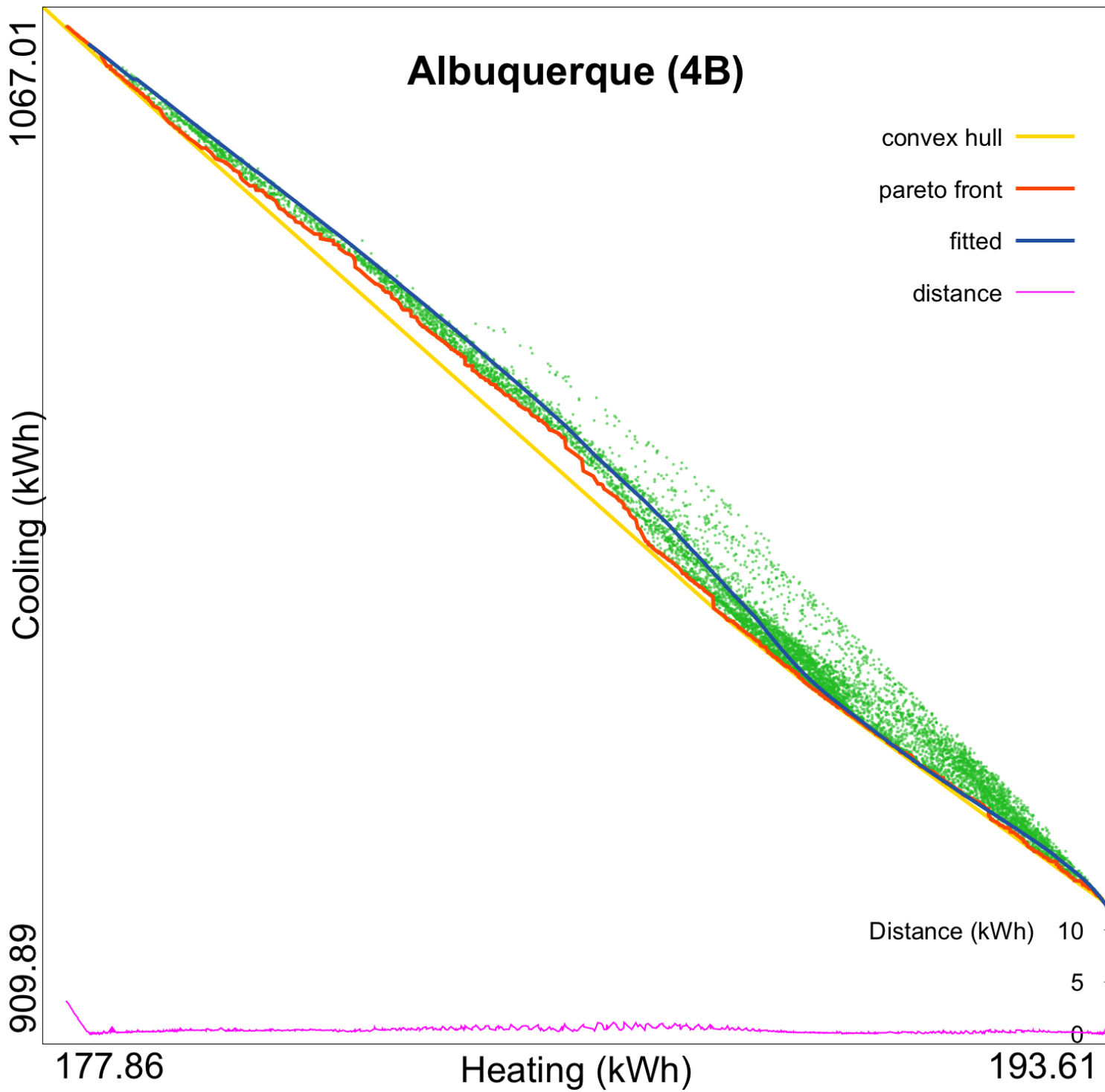

# Baltimore (4A)

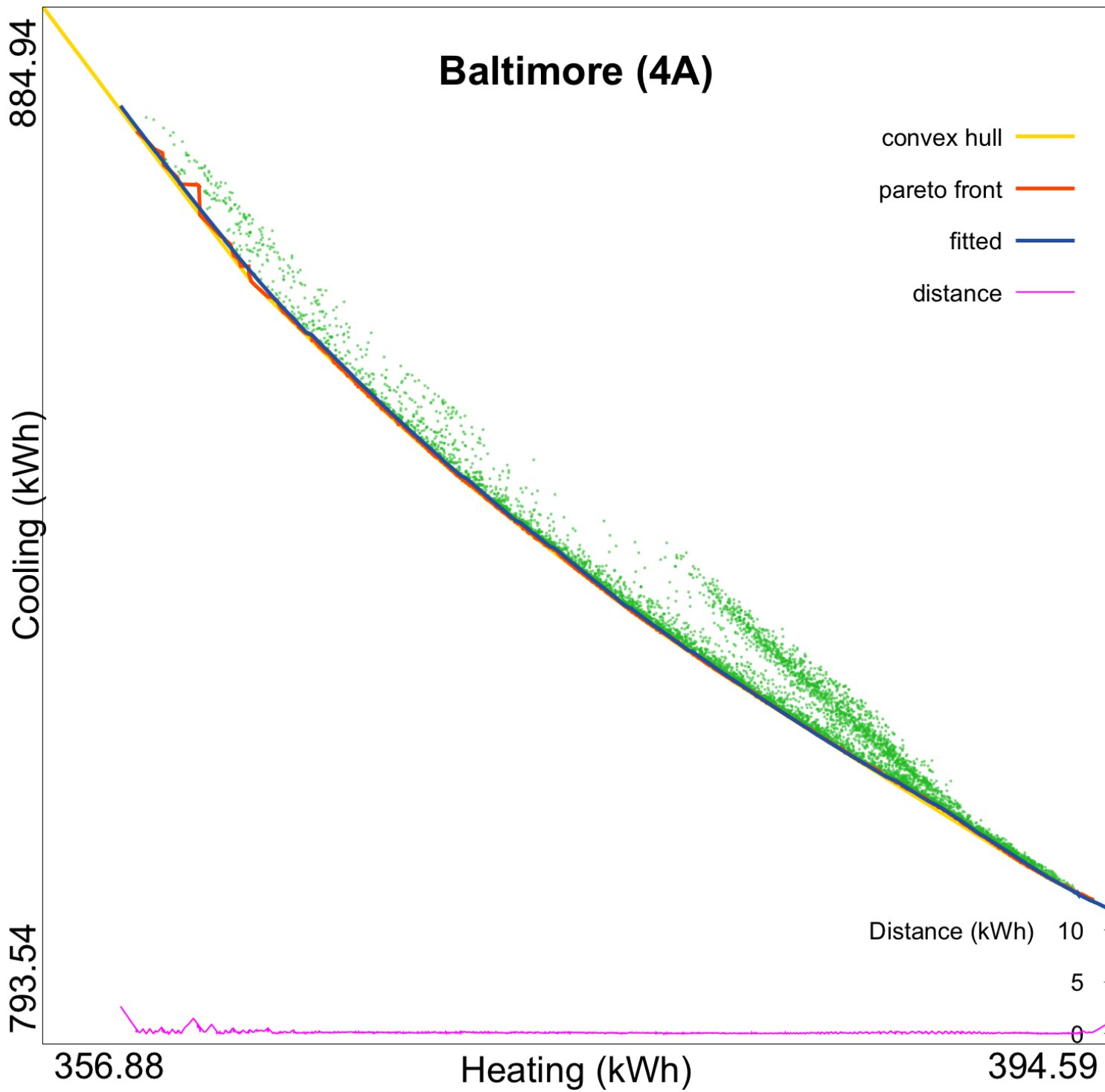

# Boise (5B)

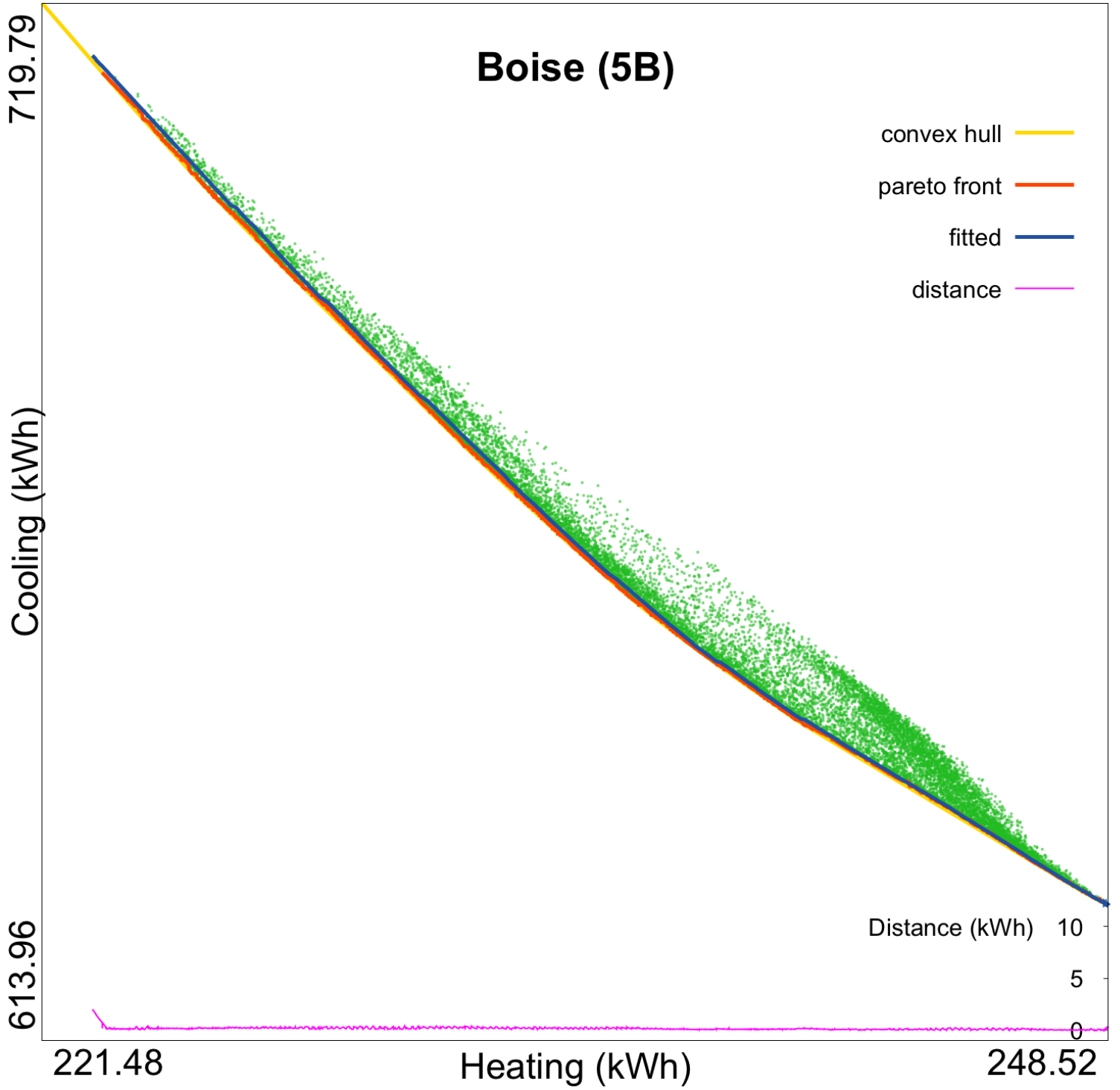

# Burlington (6A)

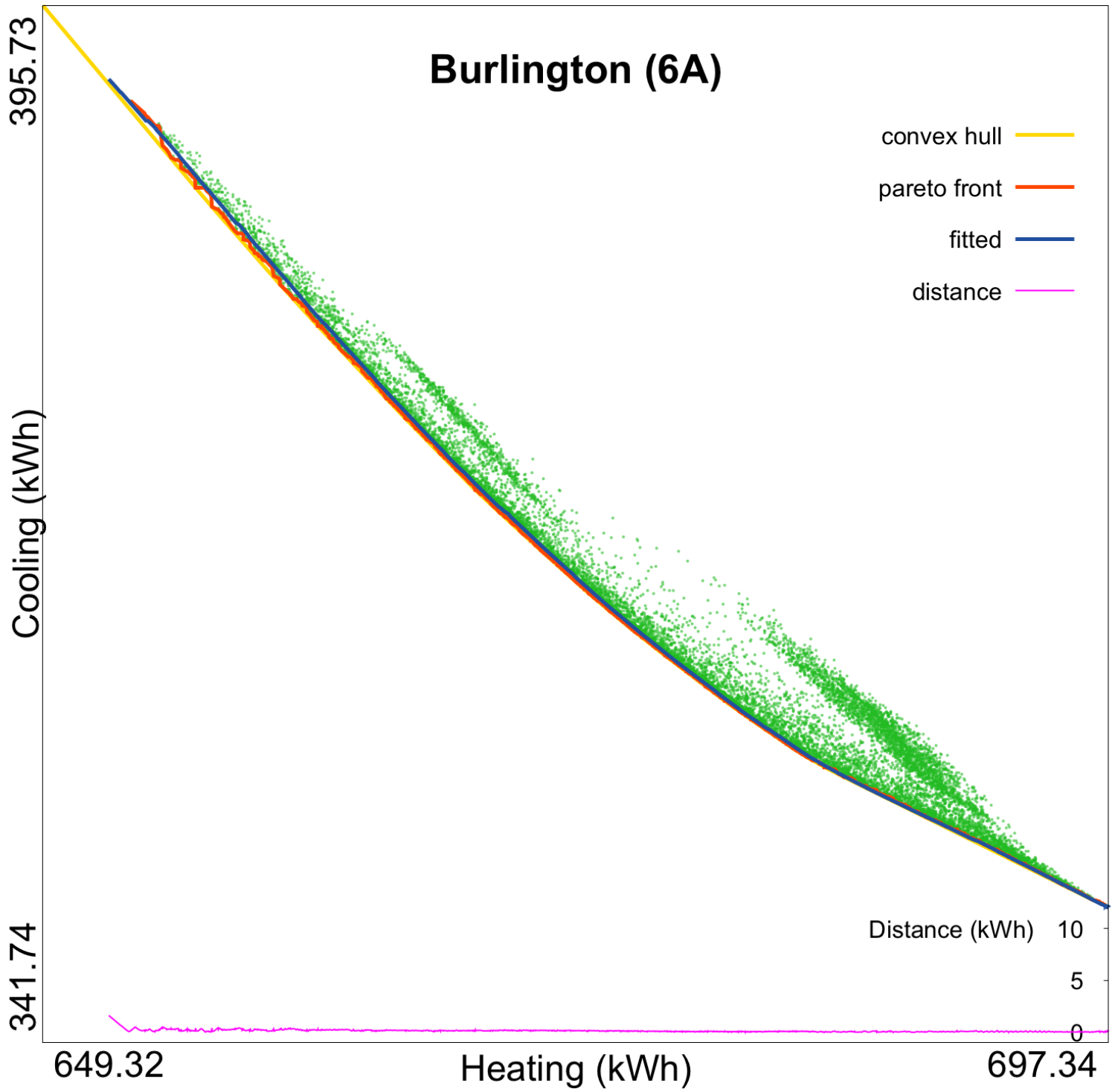

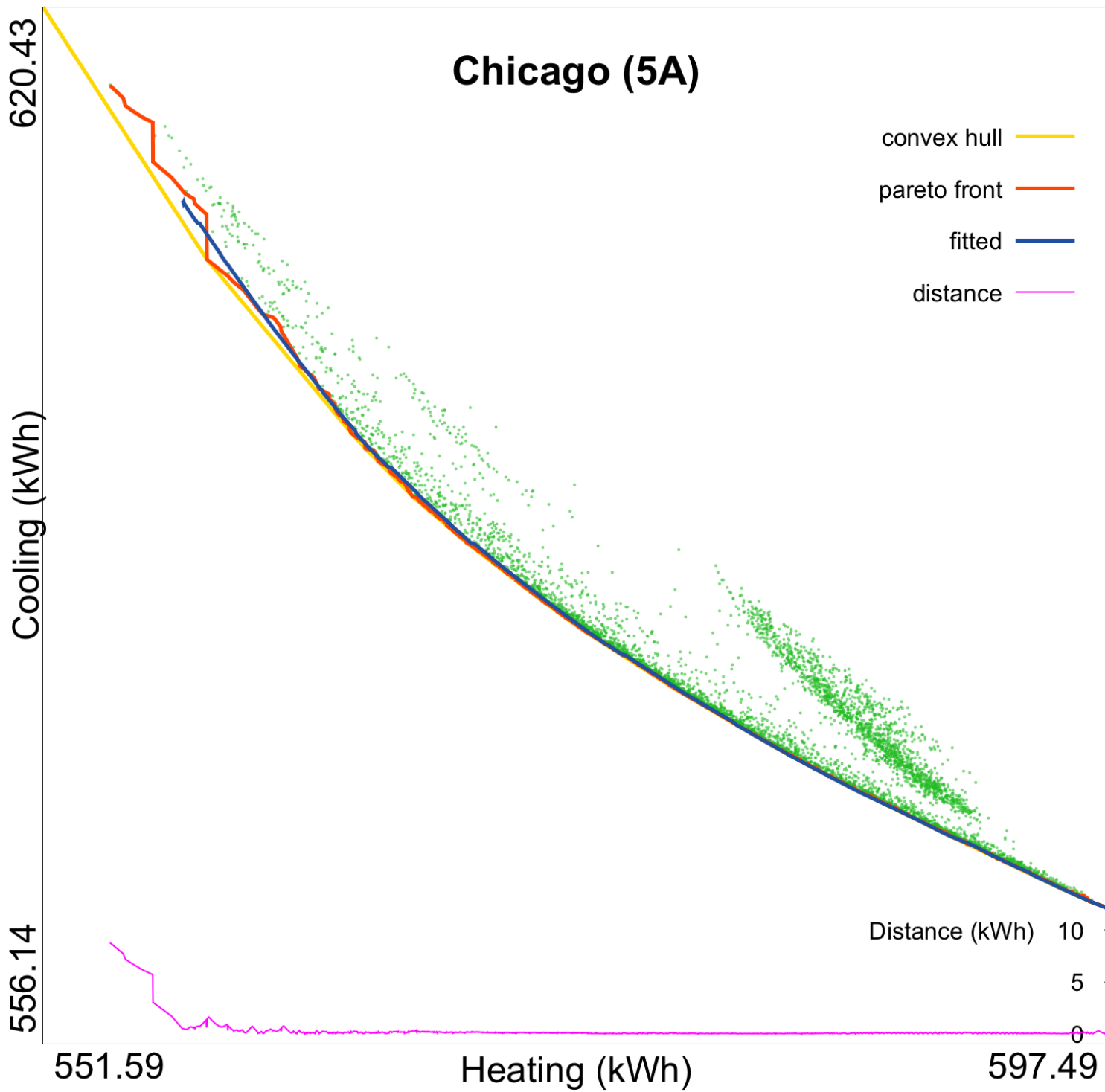

# Duluth (7)

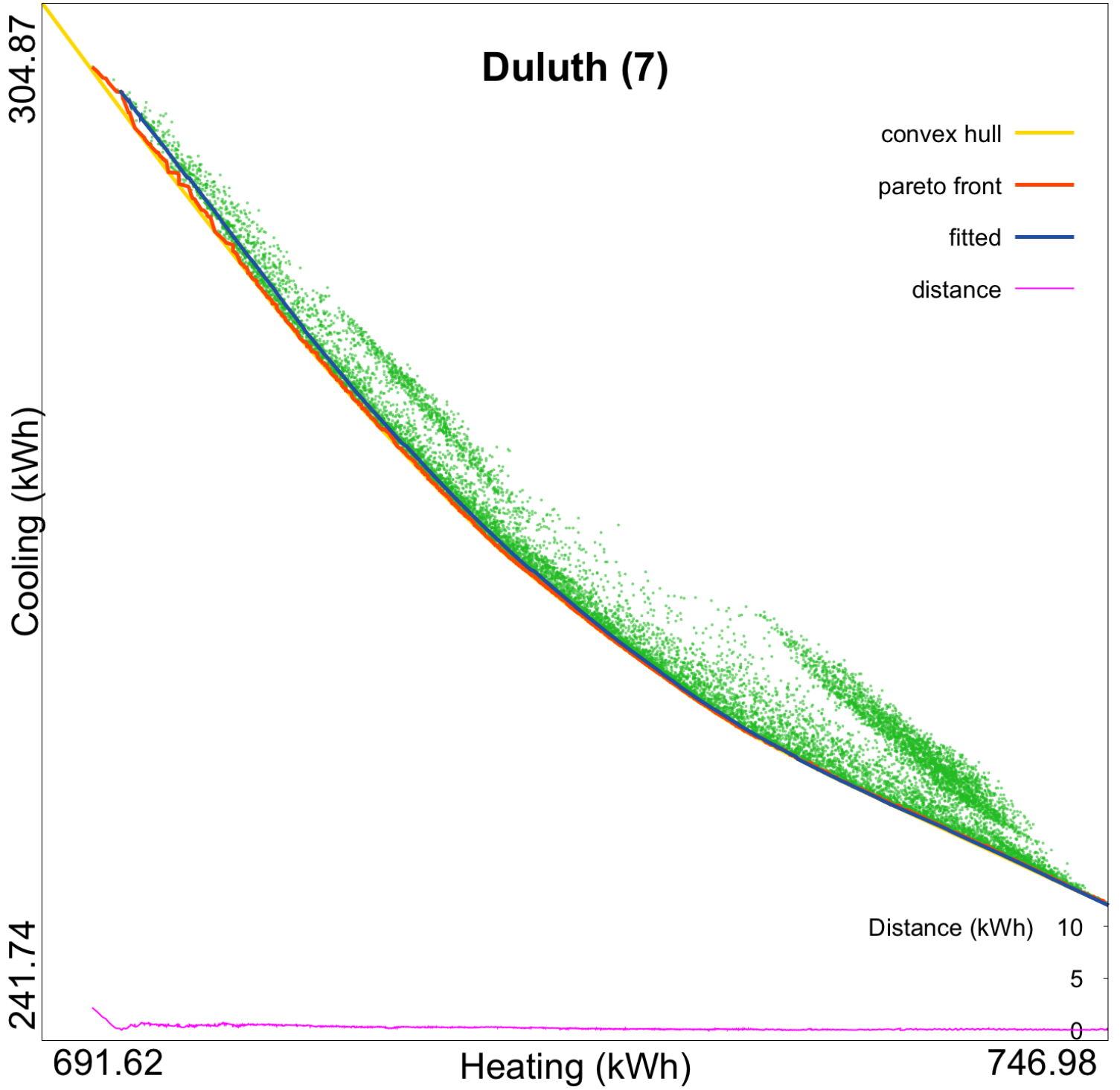

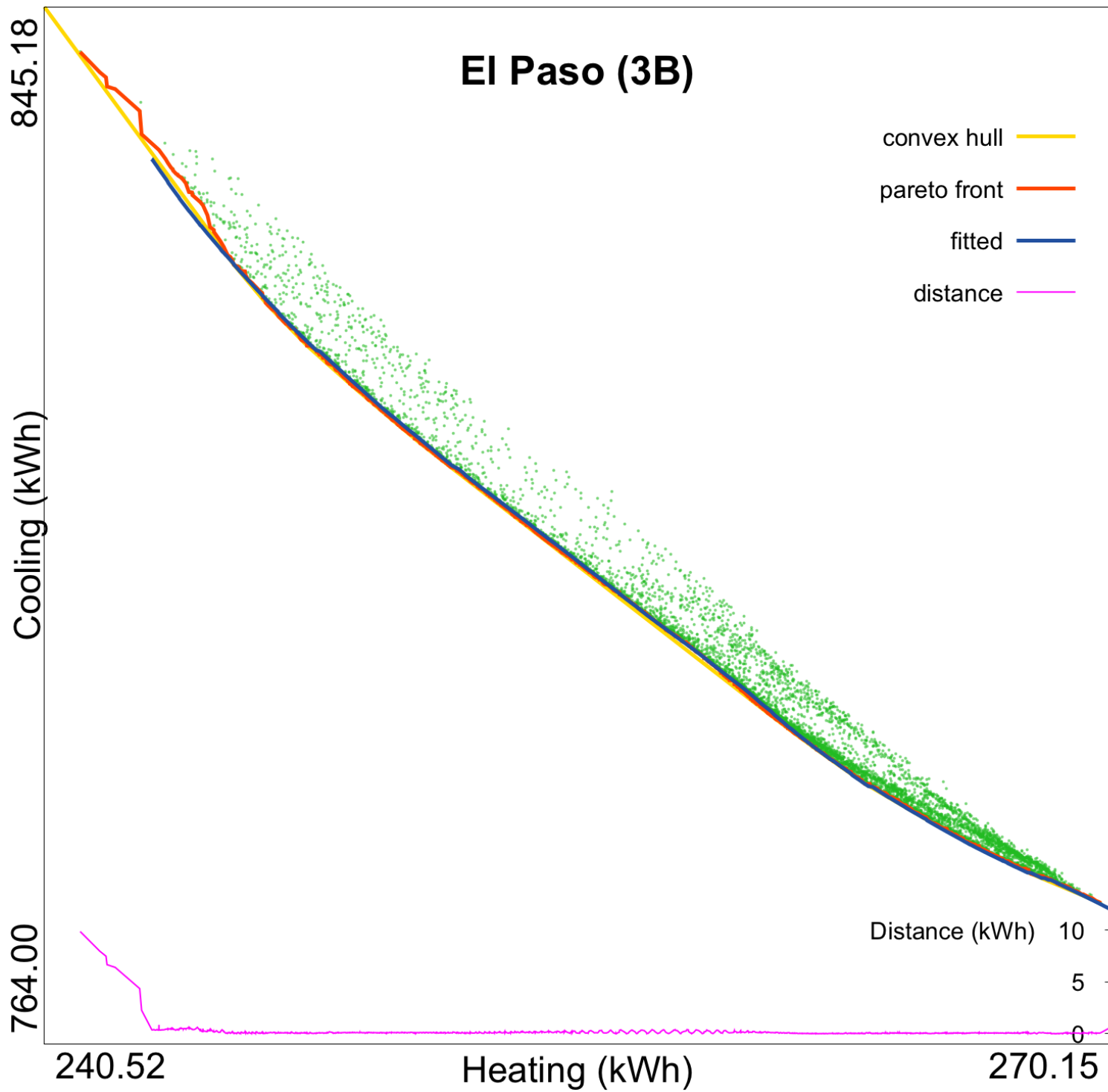

# Memphis (3A)

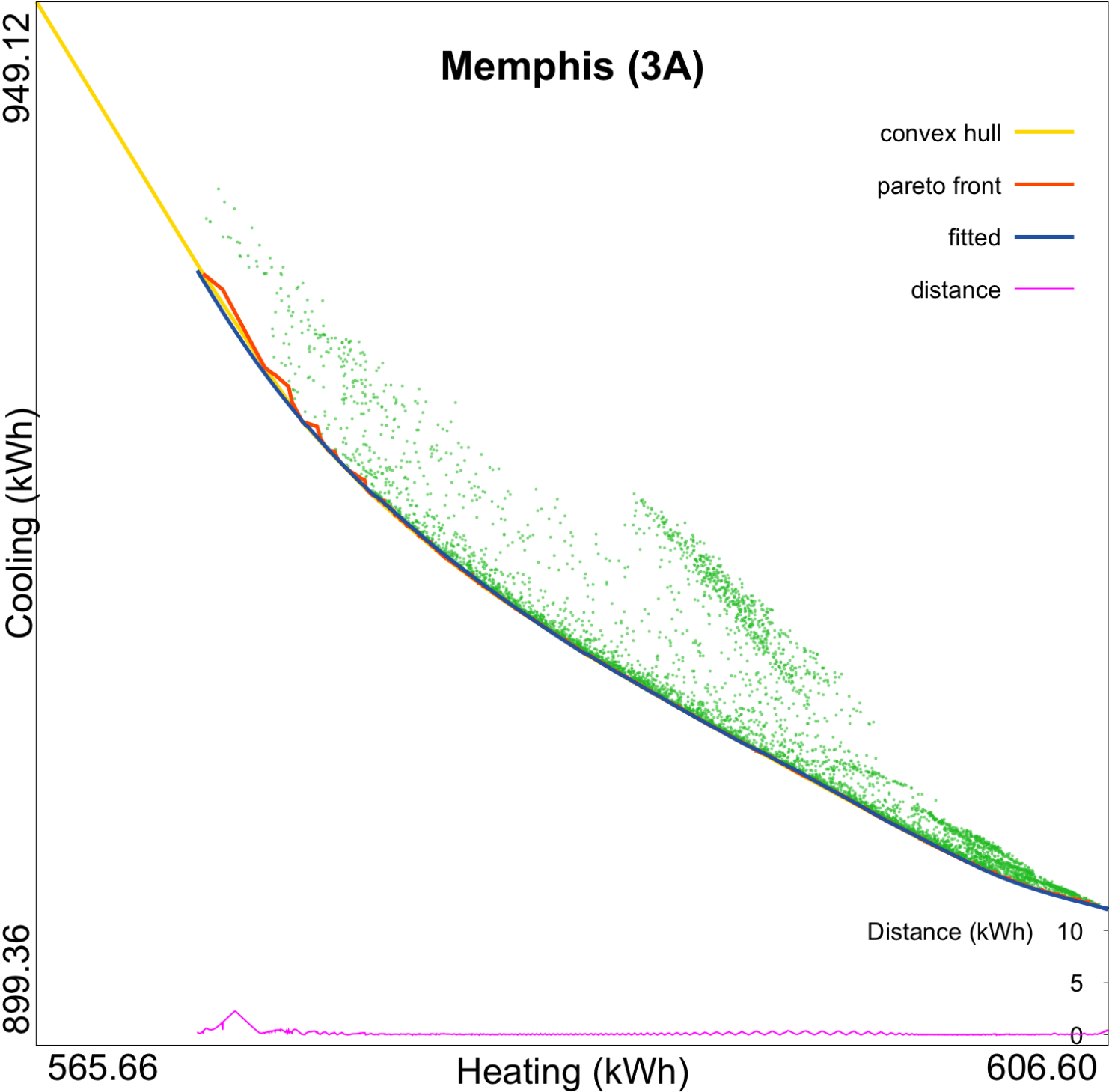

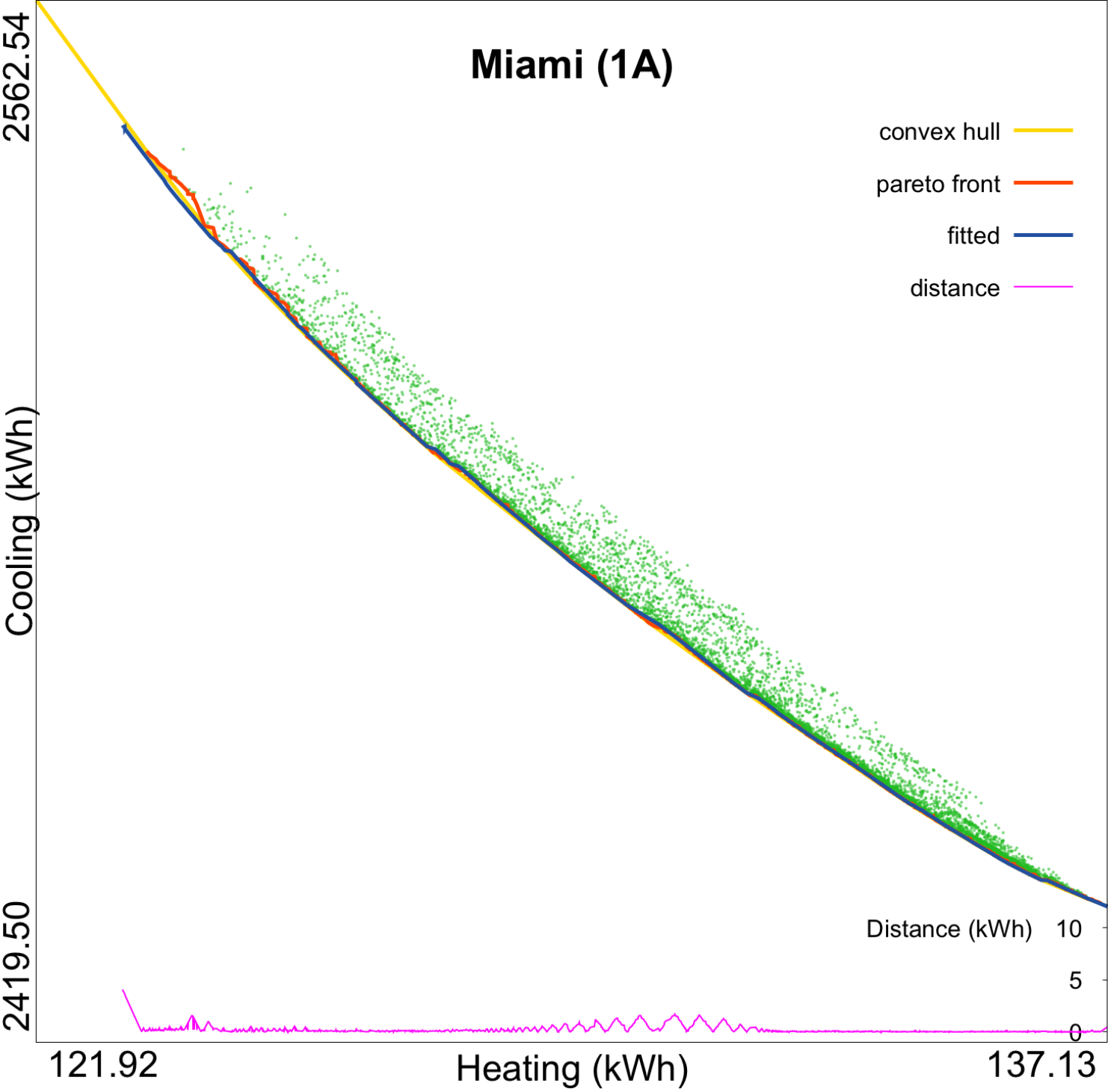

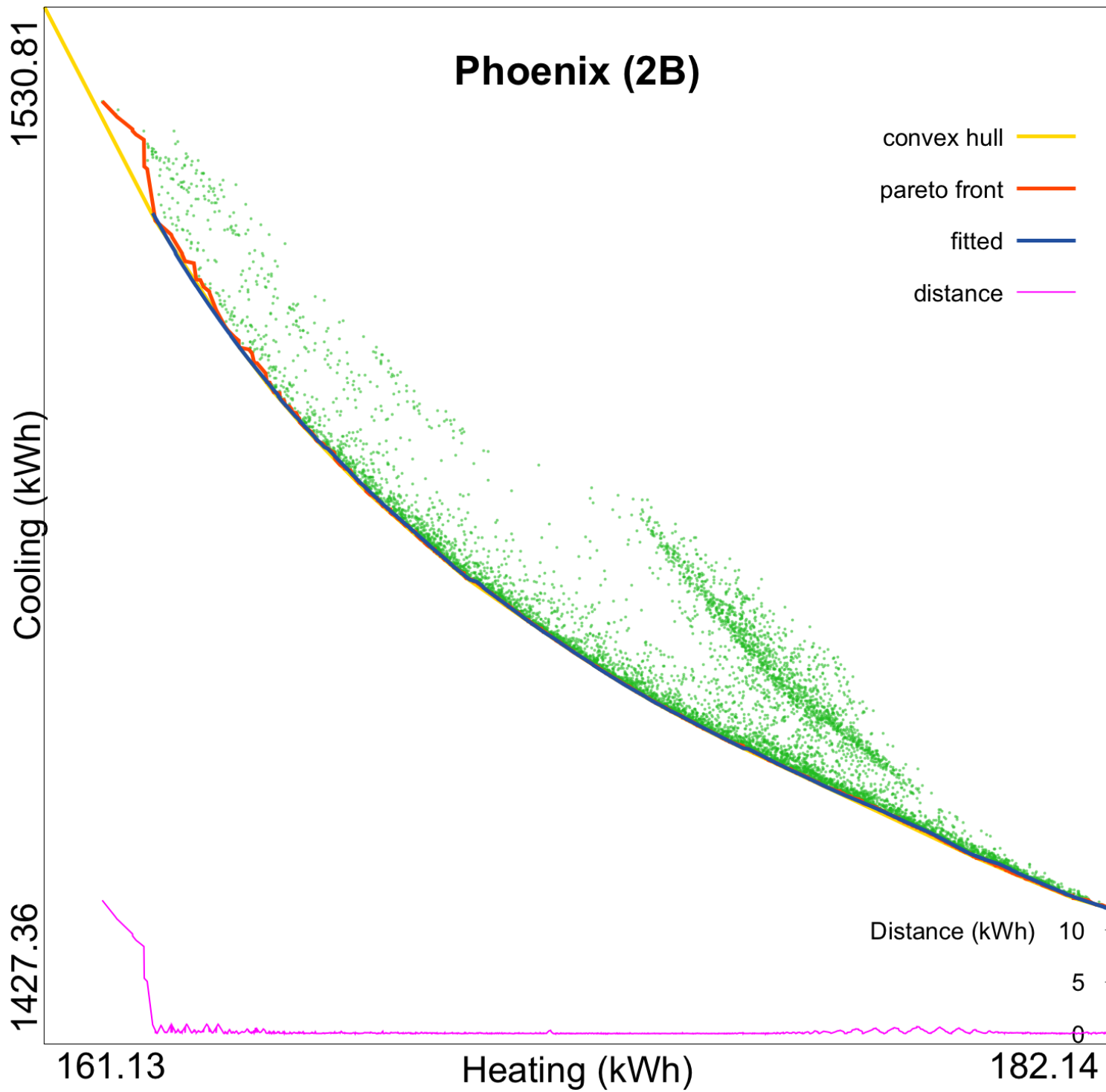

# Salem (4C)

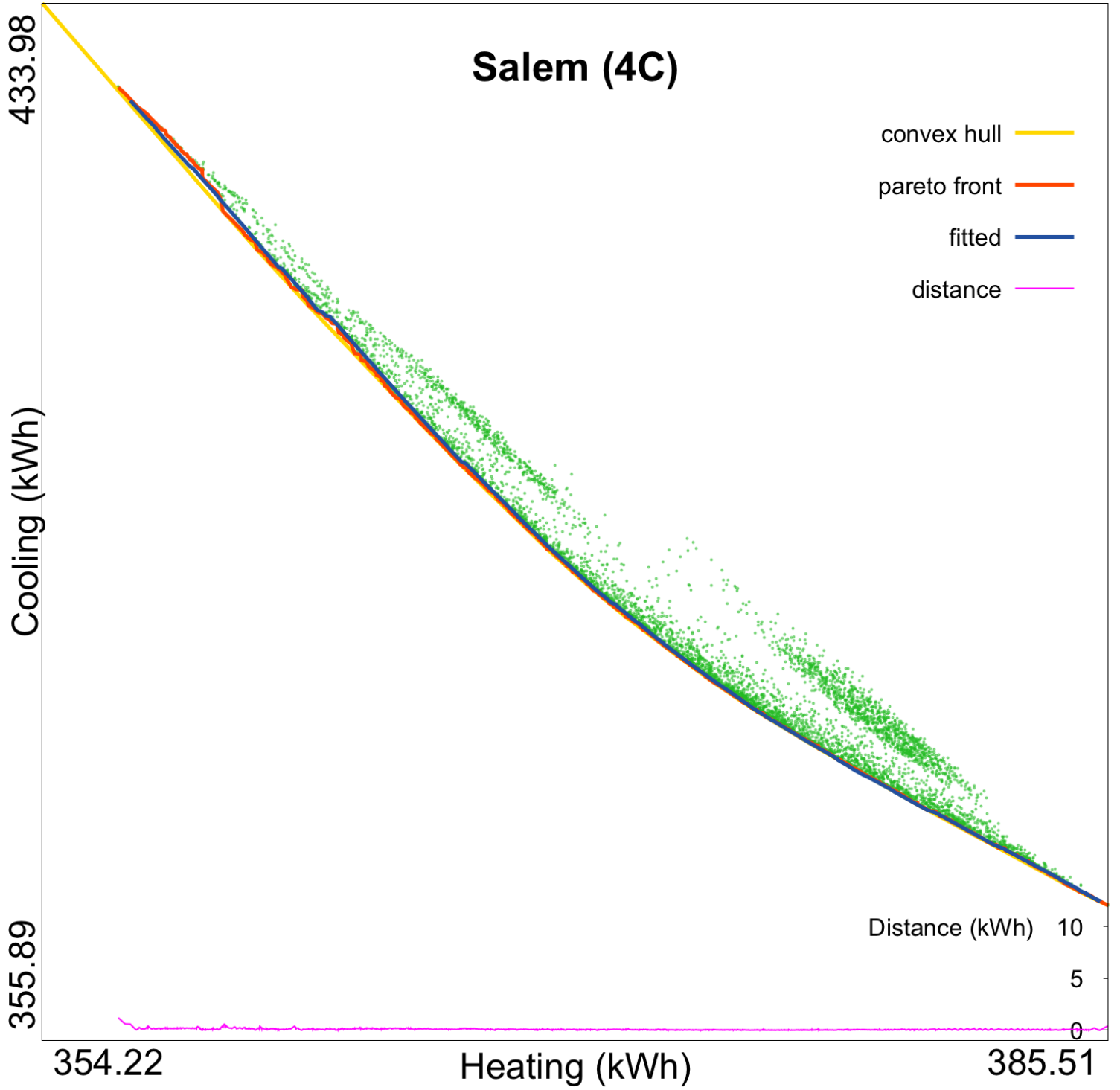

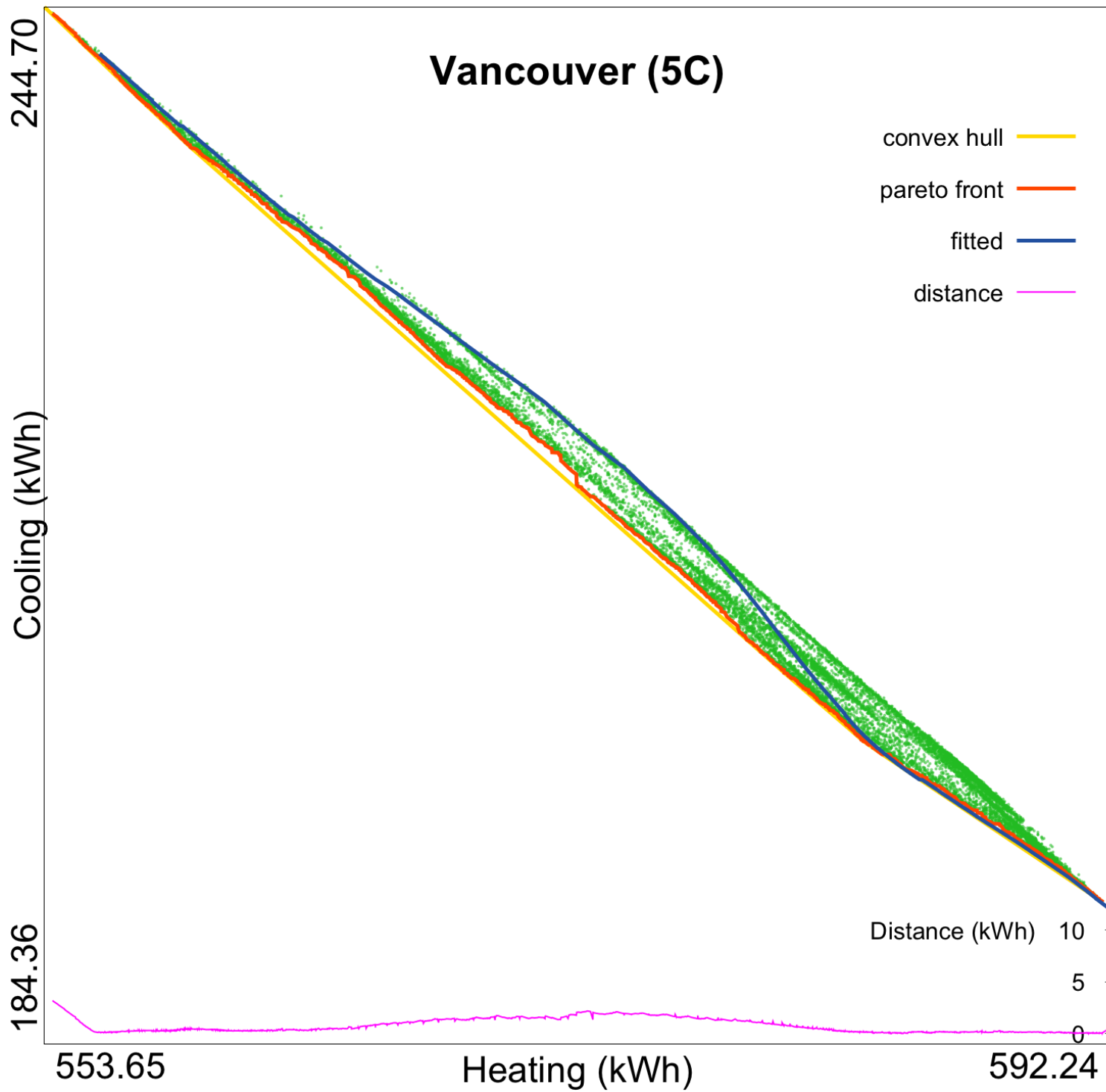

# Fairbanks (8)

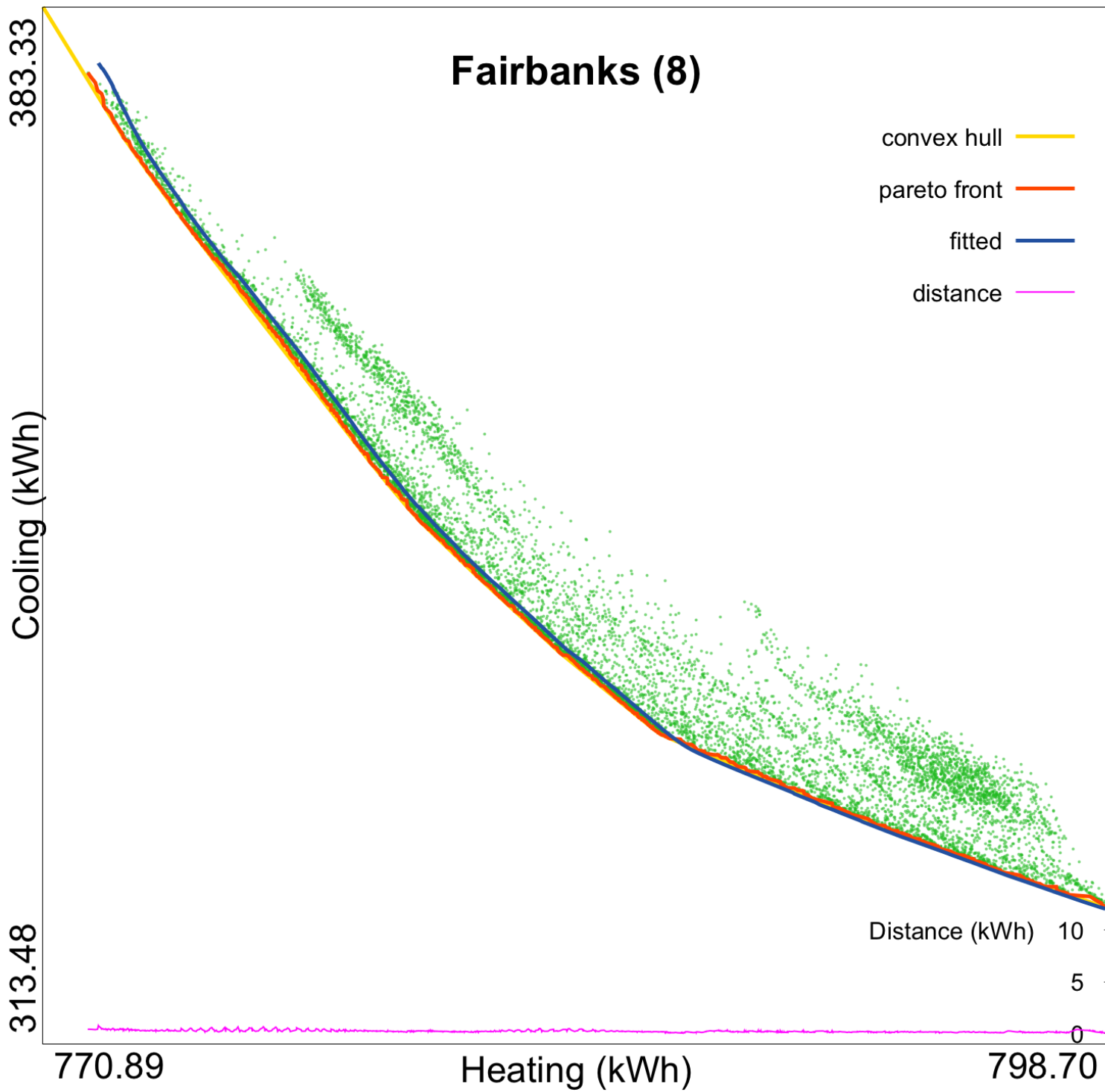

# Helena (6B)

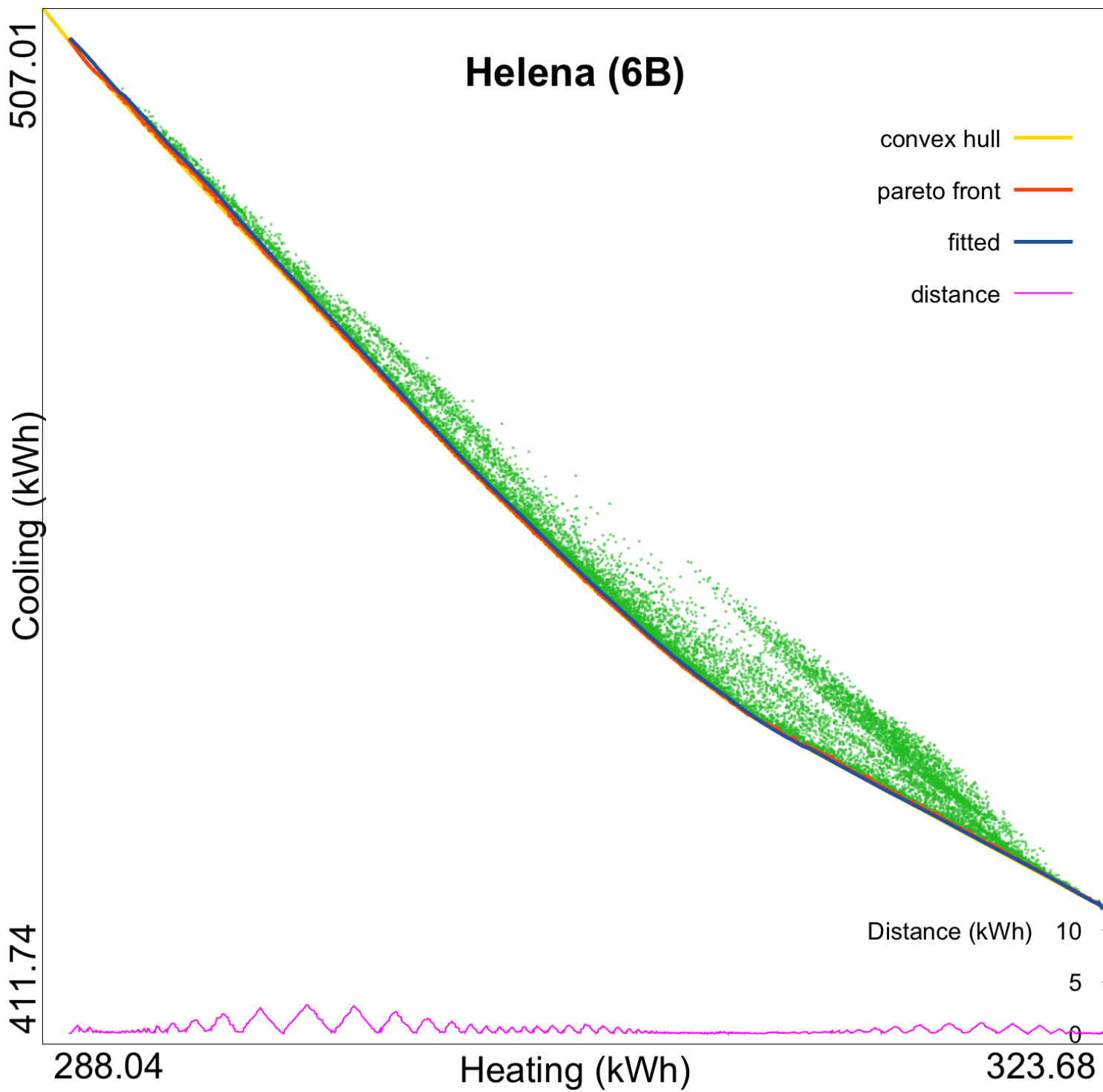

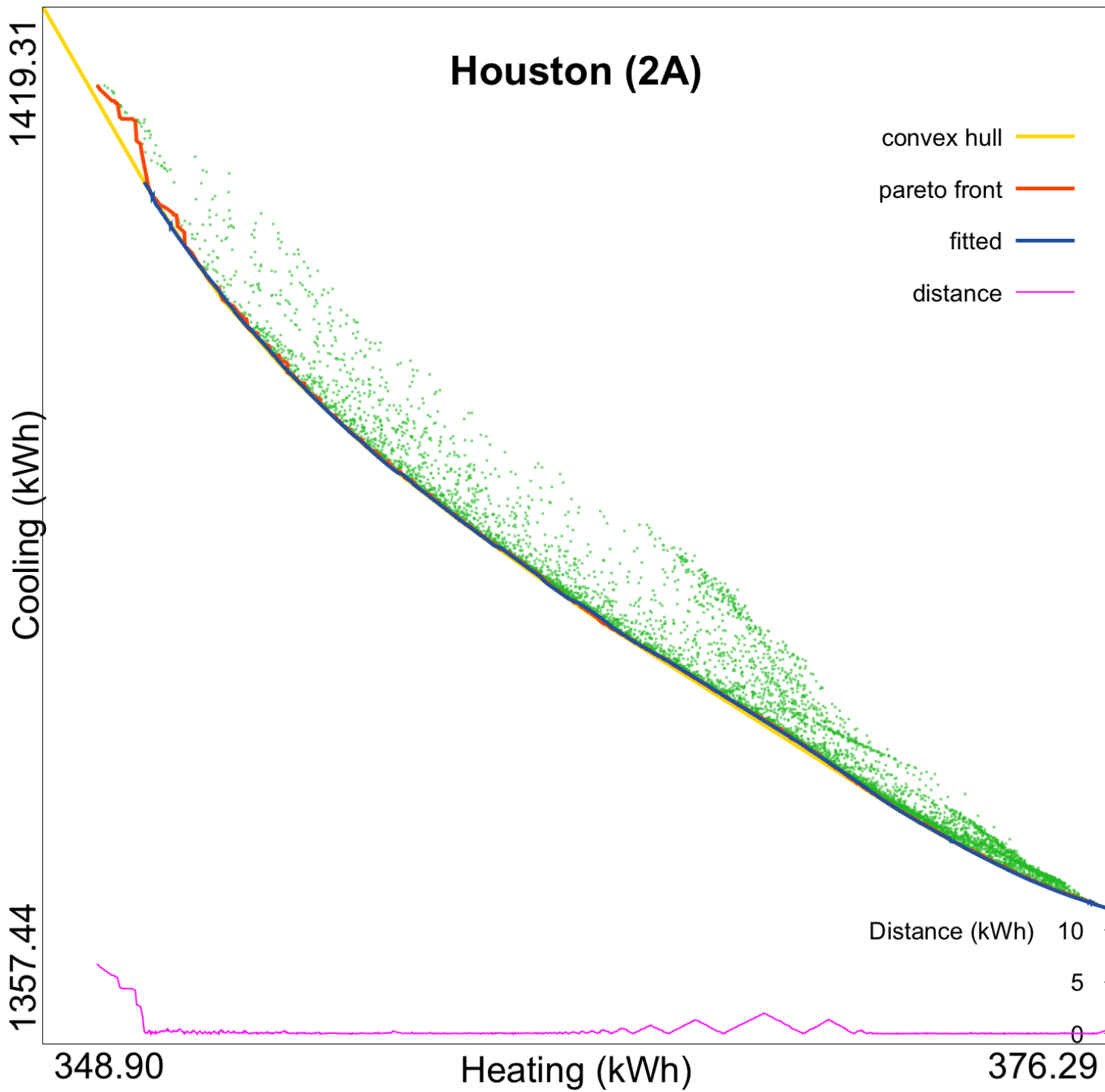

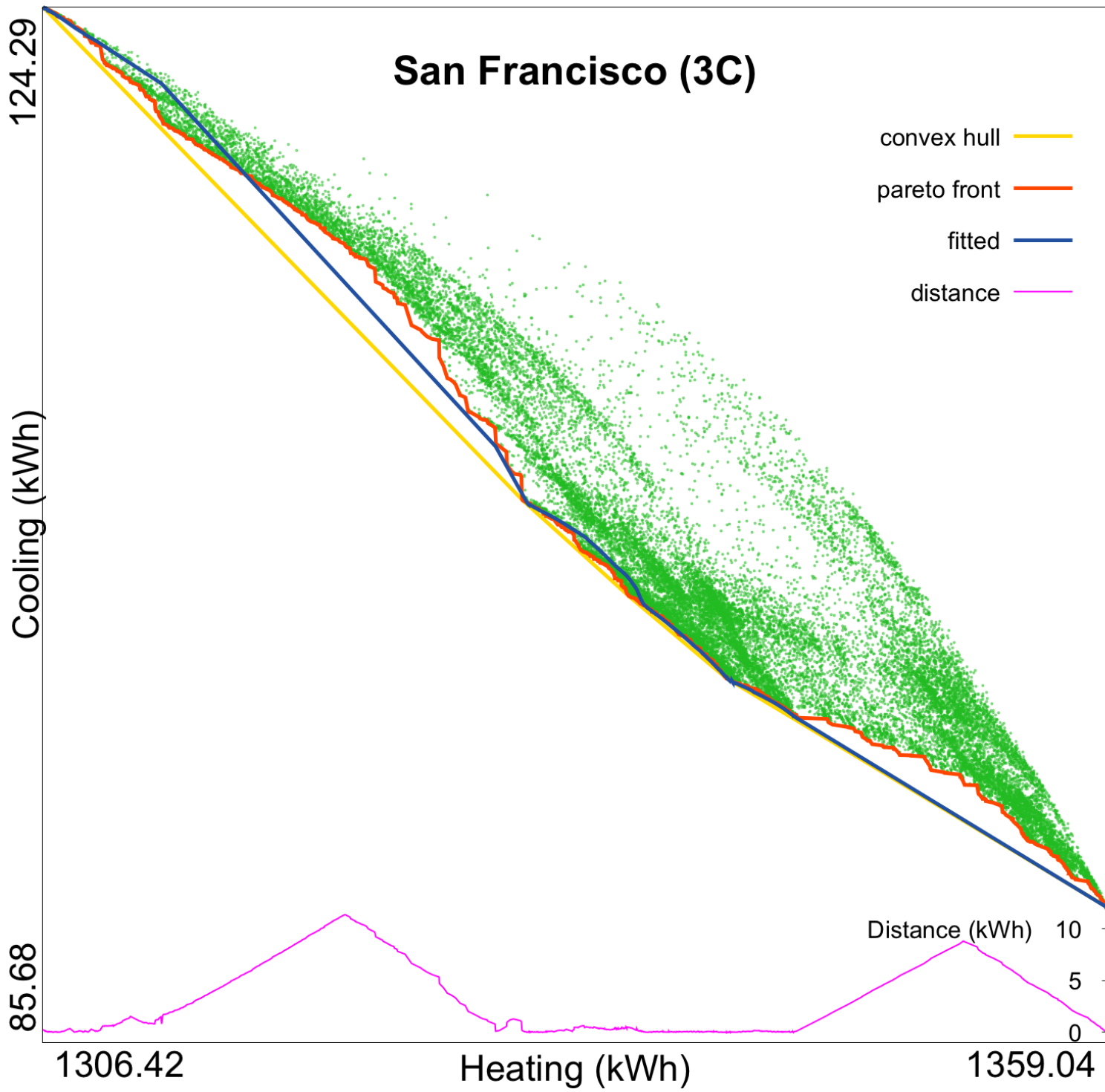

Supplement: S2 File — (PDF) [file pone.0212710.s002.pdf]
